# Margin of Error: The Emerging Role of Field Cancerization in Predicting Recurrence Risk of Ductal Carcinoma In Situ

**DOI:** 10.3390/ijms27062523

**Published:** 2026-03-10

**Authors:** Sophia Hu-Lieskovan, Olivia Banks, Rose Davidson, Dana Franklin, Padmashree Rida, Nikita Jinna

**Affiliations:** 1 School of Biological Sciences, University of Utah, Salt Lake City, UT 84112, USA; u1297691@utah.edu (S.H.-L.); olivia.banks@utah.edu (O.B.); 2 Department of Biology, Westminster University, Salt Lake City, UT 84105, USA; mjd0531@westminsteru.edu; 3 City of Hope Comprehensive Cancer Center, Duarte, CA 91010, USA; dfranklin@coh.org; 4 Science Department, Rowland Hall, Salt Lake City, UT 84102, USA; padmashreerida@rowlandhall.org

**Keywords:** ductal carcinoma in situ, ipsilateral recurrence, field cancerization, ductal remodeling, exosomes, tumor margin, histologically normal

## Abstract

Although ductal carcinoma in situ (DCIS) diagnoses continue to climb, patient management remains constrained by limitations in recurrence prediction. Conventional histopathology and existing prognostic parameters often inadequately predict local recurrence, leading to over- or under-treatment. Additionally, discourse remains over the clinical implications of margin width as a measure of recurrence risk, demonstrating the limitations of a margin-based model, and motivating our proposal that recurrence risk is dynamic and should be defined by patient-specific, spatially resolved diagnostic biomarkers. This review introduces field cancerization as a framework that may illuminate mechanisms underlying DCIS ipsilateral recurrence and improve clinical decision-making. We propose that the potential drivers of ductal field cancerization span two stages: pre-tumorigenesis and post-tumorigenesis. Pre-tumorigenic events include non-biological and biological exposome factors. Post-tumorigenic drivers include intratumoral and microenvironment-mediated remodeling of adjacent tissues that promote malignancy. This review bridges stage-specific molecular mechanisms to potentially actionable strategies for DCIS patient management—particularly margin assessment and recurrence risk prognostication—while highlighting the critical unmet need to identify biomarkers that measure high-risk field changes. We also emphasize the need to move beyond lesion-centric management toward multivariable prognostic models that include distance-mapped field biomarkers, enabling more precise surgery, improved selection of adjuvant therapy, and safer de-escalation for low-risk patients.

## 1. Introduction

Prior to the early 1980s, ductal carcinoma in situ (DCIS), known then as non-invasive intraductal carcinoma, was uncommon, but treatment was straightforward. Patients were treated with a mastectomy, and the prognosis was positive [1]. However, with concerted community educational programs, advancements in screening and detection, and the advent of deep learning-based algorithms, rates of DCIS diagnoses have drastically increased in recent decades [2]. In 2022, approximately 51,400 new cases of DCIS were documented [3]. The estimate for 2024 increased to 56,600 new cases [4]. Thus, the urgency of developing effective DCIS management strategies becomes more apparent with each year.

Surgical resection is the current gold standard treatment for managing DCIS [5]. This treatment modality includes breast-conserving surgery (BCS) and mastectomy, depending on disease severity and stage. Although adjuvant radiation therapy (RT) does not impact survival rates, it is often included in treatment strategies in addition to BCS. However, RT is not common post-mastectomy, and remains contested as a necessary treatment given its association with increased risk of secondary non-breast cancers, ischemic heart disease, and other negative effects [6,7,8]. A 2015 study found that most DCIS patients remain free of recurrence, with 12-year ipsilateral recurrence rates at 7.5% and 13.4% for low-intermediate and high-grade disease patients, respectively [9]. Given that DCIS itself is not life-threatening, common treatment approaches—particularly the frequent use of adjuvant RT post-BCS—and its description as “early breast cancer” have been called into question. Rather, the importance of identifying patients with a high risk of ipsilateral recurrence, who need more intensive adjuvant treatment, has been emphasized.

Multiple groups have proposed tools for evaluating recurrence risk. In 2010, researchers at the Memorial Sloan Kettering Cancer Center developed a nomogram that integrates 10 clinicopathologic variables—age at diagnosis, breast cancer (BC) family history, clinical/radiologic presentation, type of adjuvant treatment if applicable, nuclear grade, presence of necrosis, status of surgical margins, number of surgical excisions, and year of surgery—to calculate individualized risk estimates [10]. In 2010, Milonas et al. provided external validation of the afore-mentioned nomogram, determining accuracy as between 74.4% and 86.2% [11]. The Risk-of-Recurrence Score derived from the Oncotype DX DCIS test is another gene expression-based evaluative tool that predicts 10-year risk of recurrence and efficacy of adjuvant radiation therapy [12]. Another evaluation lies in the recent development of DCISionRT^®^, a gene expression-based biosignature that evaluates recurrence risk, which has enhanced the reliability of assessing the need for radiation therapy and has been shown to potentially reduce unnecessary radiation in patients with low risk [13]. However, despite achieving Advanced Diagnostic Laboratory Test (ADLT) status, this biosignature has not yet received FDA approval and therefore has not been widely adopted in routine clinical practice.

In breast-conserving surgeries, the clinician locates the tumor and removes it, along with a margin of varying width. However, despite the presence of clear margins—negative for cancer cells—after resection, 10–50% of BCS patients require a second operation (Re-Breast Surgery, or RBS) and local recurrence continues to occur at 13–25% of patients [14,15]. Patients treated with mastectomy for DCIS have a 10-year recurrence rate typically in the range of 1–5%, with recurrences more commonly presenting as invasive disease [16]. Thus, margin width, perhaps the only clinically modifiable variable influencing ipsilateral recurrence, has become a topic of critical discussion within the BC management community. Despite the 2 mm margin joint recommendation by The Society of Surgical Oncology, American Society for Radiation Oncology, and American Society of Clinical Oncology, a consensus for margin adequacy does not exist for DCIS treated with BCS and whole-breast radiation. Thinner margins are preferred for a shorter recovery time, fewer complications during post-operative recovery, aesthetics, and quality of life. In support of excising smaller margins, a recent study from Co et al. found that close surgical margins (less than 2 mm) were not associated with increased risk for local recurrence in DCIS [16]. Schmitz et al. also found only a weak association between DCIS size and margin status and the risk of subsequent invasive BC in the same breast [17]. However, as an apparent contradiction to what the aforementioned studies concluded, mastectomy was found to be associated with a lower recurrence rate than BCS in a study by Chien et al., suggesting that larger resections result in a lower likelihood of recurrence [18]. These disparate results raise the question of whether inherent spatial and molecular differences exist in the characteristics of cells in tumor margins between different patients, and whether a “universal” margin width recommendation should even exist. As the clinical management of DCIS continues to evolve, it is crucial to explore the underlying mechanisms that contribute to recurrence, which may lie in the broader concept of field cancerization. In the context of DCIS, and indeed, oncology at large, field cancerization refers to the concept that recurring ipsilateral tumors arise from a field of pre-neoplastic genetically and/or epigenetically altered cells that predispose an entire region to tumorigenesis [19]. The field itself may not exhibit obvious histological abnormalities, meaning that the aberrations within these cells may remain undetected by clinical breast examination, mammography, or ultrasound. Historically, the concept of field cancerization was specifically applied locally to tissue fields that were directly exposed to carcinogens, such as cigarette smoke or UV light (e.g., the epithelium of the aerodigestive tract or UV-damaged skin). However, field cancerization can be applied more broadly to all endogenous and exogenous factors that engender a population of genetically and/or epigenetically altered cells with an increased risk of cancer development (such as the entire body, in the case of hereditary cancers, or cells in peri-tumoral margins, that may come under the influence of the tumor). This perspective is more holistic and acknowledges the potential for a multifactorial nature of field cancerization. This article examines field cancerization of the breast that may arise both pre- and post-tumorigenesis. The former refers to conditions and factors that exist prior to the genesis of a primary tumor and ultimately predispose the region to tumor recurrence, while the latter category refers to field cancerization, after and as a result of the primary tumor.

## 2. Breaking New Ground: Ductal Carcinoma In Situ to Invasive Ductal Carcinoma

DCIS refers to the non-invasive proliferation of neoplastic epithelial cells within the ducts of the breast [20,21]. Although non-invasive, DCIS is seen as a potential precursor to invasive ductal carcinoma (IDC), in which neoplastic epithelial cells escape ductal confinement. Compared to the 10-year age-adjusted risk of a less than 3% chance of a new DCIS diagnosis, the risk of a recurrent ipsilateral DCIS or IDC diagnosis is considerably higher following a DCIS diagnosis [22]. However, it has been suggested that DCIS associated with IDC may evolve through different pathways than pure DCIS (associated with no invasiveness). An analysis of 28 different cases found that pure DCIS cases show a greater loss of heterozygosity (LOH) rate in the margins than the DCIS cases associated with IDC for 11 out of 15 microsatellite markers [23]. Two of these markers, D13S260 and D17S800, map closely to the BRCA1 and BRCA2 genes, suggesting that these BC susceptibility genes may play a role in tumorigenesis [24].

Fragmentation of the myoepithelium and the basement membrane often occurs as a result of the DCIS to IDC transition. The loss of these physical barriers allows tumor cell invasion into the microenvironment and lymphovascular space. Histologically normal myoepithelial cells perform several tumor-suppressive roles; however, before the myoepithelial cell layer is broken through, DCIS-associated myoepithelial cells exhibit changes in the expression of structural proteins such as calponin, CD10, and smooth muscle myosin [25,26,27]. Additionally, myoepithelial cells adjacent to DCIS lesions show increased expression of proteins that promote extracellular matrix (ECM) remodeling and decreased expression of proteins that inhibit ECM remodeling [28]. Mammary fibroblasts are progressively altered during malignant progression and show an increase in the expression of ECM remodeling proteins such as the collagen alpha-1(XI) chain, prolyl 4-hydroxylase A2, and cathepsin V. Fibroblasts in DCIS lesions adjacent to invasive BC show significantly higher levels of these proteins compared to the fibroblast surrounding pure DCIS lesions, suggesting increasing expression of these proteins begin in the pre-invasive state [28,29,30].

## 3. Ring of Fire: Field Cancerization

To better understand the role of field cancerization in local recurrence, it is vital to examine existing models of DCIS progression, including how different mechanisms of recurrence are defined. As developed in further sections of this review, local recurrence after BCS can arise from residual disease, clonally related recurrence, or from an independent second primary tumor that arises within the predisposed field but is genetically distinct from the original tumor. These mechanisms provide a framework for interpreting evolutionary models of tumor progression and recurrence.

In recent years, four dominant evolutionary models for DCIS progression have emerged, tracking the progression from normal ductal tissue to DCIS, and then from DCIS to IDC [31]. All of these models either support or are compatible with the conceptual framework of field cancerization.

The first evolutionary model, known as the independent lineage model, proposes that in situ and invasive cell populations arise independently within a population, and do so in parallel. A recent study found molecular differences between these sub-populations, which supports this theory [32]. Another study from Casasent et al., however, found that DCIS and IDC share a common lineage [33]. Neither of these findings is at odds with the notion of field cancerization, as a field of cells predisposed to develop tumors is not necessarily homogeneous and—as described in the sections of this review that follow—may in fact exhibit substantial heterogeneity owing to genomic instability. It would therefore be conceivable that clones of cells that develop into in situ tumors and invasive ones may arise independently of each other and in parallel, from such a field. The second evolutionary model, known as the evolutionary bottleneck model, suggests that while individual cells within a duct may develop various mutations, only a select subpopulation with a particular genotype possesses the ability to invade into adjacent tissue. In support of this model, a study from Wang et al. found that the same chromosome abnormalities were found in both the primary DCIS tumor and recurrent tumors [34]. This model most directly supports the field cancerization model, as it suggests that both the primary tumor and the recurrent one can trace their origins to a common clone in a pre-existing cancerous cell field. The third evolutionary model, known as the convergent phenotype model, suggests that diverse genotypes of DCIS cells can result in invasive tumors that exhibit the same phenotype. This model does not directly support or contradict the field cancerization theory, but instead provides a perspective that is compatible with it. The fourth evolutionary model, known as the multiclonal invasion model, proposes that multiple clones can escape ducts and invade adjacent tissues, contributing to the development of IDC. This model supports field cancerization, proposing that distinct clones can appear from more than one region within the duct. Collectively, all four of these evolutionary models demonstrate that DCIS development and recurrence are governed by spatially and biologically diverse cell populations rather than simply uniform lesions with clear margins, reinforcing the need for integrative frameworks that link field biology with clinical risk stratification.

Although this article focuses on the potential role of field cancerization in progression from normal duct to DCIS, and the risk of ipsilateral recurrence after removal of the primary tumor, we assert that the ideas proposed herein are applicable more broadly to BC, and perhaps even to other solid tumors. The upcoming section of our article, centered on pre-tumorigenesis drivers of field cancerization, will examine an array of factors and exposures occurring prior to tumorigenesis that could contribute to both the risk of developing the primary tumor as well as subsequent ipsilateral tumors. The subsequent section (post-tumorigenesis) will focus on the molecules and signals emanating from the primary tumor that could impact the risk of ipsilateral recurrence. The final section of this article will synthesize these concepts into integrative frameworks that link field biology with clinically actionable risk stratification, while also addressing limitations in the current literature and the research implications of field cancerization.

## 4. Pre-Tumorigenesis: Drivers of Field Cancerization

### 4.1. Non-Biological Drivers

#### 4.1.1. Endocrine Disruptors

The phrase “Endocrine disruptors” was first coined in 1991 at the Wingspread Conference and has since gained extensive relevance in BC research. Many have speculated that the rise in BC incidence and recurrence in the past half-century is linked to the parallel increase in prevalence of and exposure to environmental endocrine disruptors [35,36]. These chemicals can contribute to field formation by disrupting hormone signaling pathways, especially those triggered by estrogen, which plays a critical role in DCIS field formation. For instance, chemicals like bisphenol A (BPA), phthalates, and certain pesticides have been shown to bind to estrogen receptors and activate pathways in breast cells that can promote abnormal cell growth. By disrupting these pathways, endocrine disruptors may induce molecular changes that extend across the field.

Phthalates are a common plasticizer in consumer products, and have been shown to affect the incidence of BC [37]. Ahern et al. found a two-fold increase in estrogen receptor positive (ER+) BC risk (hazard ratio: 1.9) with high-level exposure to dibutyl phthalate (≥10,000 mg cumulative) [38]. A study by Segovia-Mendoza et al. that did not directly focus on DCIS recurrence suggested that phthalates interfere with anti-hormonal cancer treatments, which could feasibly be a symptom of field mutations caused by endocrine disruptions [37]. However, phthalates remain controversial as a cause of BC incidence and recurrence, as one study has conversely indicated that they can produce a protective effect, which may indicate a field-level modulatory effect [39]. The literature, thus, presents conflicting evidence regarding phthalates and BC risk. While evidence demonstrates a potential pro-tumorigenic role, the current body of evidence remains inconclusive, preventing definitive statements about their impact on BC incidence or recurrence.

Compounds in the bisphenol family have also been found to have effects on BC incidence and recurrence. These chemicals have been found to contribute to the progression of ER-positive and ER-negative BCs through different downstream mechanisms [40]. This finding supports the idea that bisphenols can induce a field of abnormal cell growth. Bisphenols have also been shown to induce inflammation, which can produce a cytokine-enriched microenvironment that may promote recurrence. This paper will discuss those effects in greater detail in the following sections. Segovia-Mendoza et al. also found that low doses of BPA can alter the proliferation of BC cells, which could potentially produce a post-tumorigenesis field effect [37]. However, the effects of bisphenols remain controversial in the context of BC incidence and recurrence.

#### 4.1.2. Diet, Alcohol, and Supplementation

Diet is thought to strongly influence the risk of recurrence in DCIS. Recent research has begun to demonstrate the importance of nutrition as a factor for field cancerization. Ethanol, a group-1 carcinogen, has been repeatedly demonstrated to increase the risk of BC [41]. In the liver, ethanol is metabolized by alcohol dehydrogenases into acetaldehyde, another well-known carcinogen. Acetaldehyde is commonly absorbed into the bloodstream, where it is quickly converted to harmless acetate. However, overconsumption of alcohol leads to an excessive buildup of acetaldehyde due to saturation of aldehyde dehydrogenases, which can lead to a toxic field effect that may increase the risk of a cancerized field. Several studies have found a correlation between moderate-to-excessive drinking and higher risk and recurrence for BC [42,43,44]. However, the effects of alcohol consumption specifically on the development of DCIS are presently unclear. In a 2010 study, Kabat et al. suggested that alcohol was not associated with risk of developing DCIS, but was more likely to affect later stages of tumor development, although the study did not investigate risk of recurrence after DCIS [45].

Soy intake has also been demonstrated to impact risk of recurrence of DCIS due to the presence of isoflavones—a natural plant-derived chemoprotectant that promotes cellular differentiation in the breast [46]. In 2009, it was discovered that recurrence risk in Shanghai women with high serum isoflavone levels had a hazard ratio (HR) of 0.77 [47]. A 2012 Nechuta et al. study combined analysis between this study and two US studies that collectively found that the HR for recurrence in women with high (above cutoff) serum isoflavone levels was 0.75 [48]. These results suggest that dietary intake of soy may be protective against the buildup of molecular alterations in the adjacent field, potentially reducing the risk for BC recurrence. Furthermore, recent research suggests that the effect of isoflavone is only significant with early exposure, which suggests that it acts as a barrier to the formation of these alterations [46]. However, the serum isoflavone levels could also influence anti-tumor immunity and other factors, reducing recurrence rates.

Given that Vitamin D has been demonstrated to reduce the overall risk of BC for years, current research has begun to focus on the role of this vitamin in recurrence. Vitamin D deficiency in Egyptian women was associated with worse disease-free survival (*p* = 0.004) for BC [49]. Similarly, multiple groups have found that women with Vitamin D deficiency had an increased risk for distant recurrence (HR = 1.94) and less favorable prognostic features, respectively [50,51]. These findings suggest that Vitamin D may play a critical role in modulating the tumor microenvironment to reduce the field effect. However, Jacobs et al. found no significant relationship between Vitamin D intake and local recurrence in BC [52]. As a result of these mixed findings, Vitamin D’s impact on local recurrence remains unclear, despite the demonstrated potential of Vitamin D in reducing distant recurrence and improving prognosis.

High glycemic index (GI) diets have also been associated with increased recurrence rate for BC, by way of elevating insulin responses [53]. While insulin is not a carcinogen at biologically normal levels, hyperinsulinemia can promote the growth of malignant cells by creating a tumor-favorable environment [54]. Insulin-induced alterations in the field could contribute to molecular changes that increase risk for local recurrence.

### 4.2. Biological Drivers

#### 4.2.1. Inflammatory Cytokines and Mammographic Density

Inflammation and breast density are also suggested to be promoters of field-driven recurrence. High body mass index (BMI) has been shown to correlate with elevated DCIS recurrence. Women in the upper decile of body mass index have twice the risk of recurrence when compared to women in the lower decile [55]. This can be attributed to the inflammatory effects of adiposity, which contributes to the creation of a pro-tumorigenic microenvironment by releasing inflammatory cytokines that promote unregulated growth and apoptosis inhibition. Many studies have documented these effects of inflammation on cancer development [56]. Takeuchi et al. directly proposes that inflammatory cytokine-enriched microenvironments may play a significant role in the development of breast tumors [57]. Additionally, another group found that inflammatory cytokines had an association with distant recurrence in HER2-negative BC [58].

The use of non-steroidal anti-inflammatory drugs (NSAIDs) as chemopreventive agents supports the idea that an inflammatory cytokine-enriched microenvironment contributes to the development of a cancerized field. In 2009, an international consensus declared the efficacy of NSAIDs in BC risk reduction, supported by numerous consequent studies [59,60]. An increase in psychological distress also supports this theory because it leads to the homeostatic production of inflammatory cytokines. Groenvold et al. found that psychological distress and recurrence in BC are positively correlated [61]. Additionally, chronic stress has been associated with general tumor development [62].

Increased mammographic density has also been found to correlate with recurrence in BC [63]. High mammographic density reflects an increased ratio of fibro-glandular components to adipose tissue, a structural shift that contributes to a cancerized microenvironment. Increased deposition of collagen and higher periductal alignment of collagen fibrils make the tissue stiffer, and this increased stiffness is postulated to be responsible for the observed increased risk of BC development [64,65]. In a 2018 review, Vinnicombe et al. stressed the importance of considering mammographic density in risk-adapted prevention and treatment [66]. Boyd et al. found that the risk of BC was four to five times greater in women with dense breast tissue [67]. Another group reported that the highest recurrence rates were seen in patients with a Breast Imaging-Reporting and Data System (BI-RADS) category of D (indicating the presence of extremely dense breasts in the patient) when compared to patients whose breasts were categorized as A, B, or C (HR = 1.84) [68]. However, the elevated risk can also be explained by the limitations of the mammogram itself, which are heightened by dense breast tissue, as dense tissue appears white and lacks contrast against potential tumors that might have otherwise been detected [69]. As mammograms of dense breast tissue have a higher potential for false negatives, women with dense breast tissue are often delayed in diagnosis [70].

A supporting factor for mammographic density as related to field cancerization is that low density is associated with low-risk populations. It has been reported that high parity is associated with low density in BC and among postmenopausal women. Both high parity and low breast density are variables associated with reduced risk of recurrence [71]. Additionally, another group discovered that women undergoing postmenopausal hormone therapy experience both higher mammographic density and higher risk for BC [72]. However, inconclusive evidence prevents definitive conclusions regarding the effects of mammographic density on recurrence risk.

#### 4.2.2. Menstrual and Reproductive Factors

Risk of DCIS recurrence in women has been linked to menstrual and reproductive factors. Many of these factors are related to the process of ductal remodeling. Ductal remodeling refers to the turnover of mammary duct cells during the menstrual cycle, which occurs in response to hormonal fluctuations [73]. This process consists of the coordinated proliferation, differentiation, and death of mammary epithelial cells. The ductal network in the breast tissue expands and reorganizes as a result of the ductal remodeling process. Throughout the course of mammary gland development, there are various stages at which this remodeling takes place naturally. These include menarche, during which the mammary glands experience significant development and expansion of the ductal network; the menstrual cycle, during which hormonal fluctuations drive the growth and regression of side branches in the ductal network; pregnancy and lactation, during which massive tissue remodeling occurs to prepare and support the mammary gland during lactation; and involution, which typically happens after weaning and restores the mammary gland to its pre-pregnancy and pre-lactation state.

Hormones operate on HR+ luminal cells during each menstrual cycle, which promotes the release of mitogenic paracrine signaling molecules. These mitogenic factors promote coordinated rounds of proliferation in basal and luminal cells, which ultimately work to induce the formation of side branches known as alveolar buds. During involution, which occurs at the denouement of each cycle, these side branches regress by means of coordinated cell death.

A hierarchy of mammary stem cells (or MaSCs) and their transient progeny can be found in the mammary glands. As MaSCs are multipotent and can self-renew, they give rise to short-lived progeny and are evenly dispersed throughout the ductal tree. The short lifespans of the progeny cells serve as a defense mechanism that ensures periodic and rapid eradication of mutant cells on a regular basis and limits the capacity for the proliferation of an oncogenic line. However, although cyclical remodeling works for the elimination of potentially harmful mutant cells, it paradoxically may promote the expansion of mutant cells that survive the aforementioned process. This can lead to the creation of large fields of cells with potentially oncogenic mutations across the epithelial network, creating a field cancerization effect.

The spread of mutant cells across the mammary epithelial network is facilitated by several interconnected mechanisms. Stochastic stem cell loss and replacement play a crucial role in this process; during each menstrual cycle, there is a random loss and replacement of mammary stem cells. Through this approach, mutant stem cells may spread and invade regions that were previously occupied by wild-type cells. Localized proliferation and apoptosis also occur due to side branch turnover during the menstrual cycle. Mutant stem cells are further encouraged to move across the ductal network by this cyclical remodeling, which gives them the chance to proliferate into recently vacated spaces.

Cooperative clonal behavior is another important mechanism that can contribute to the spread of mutant cells. Over time, the growth of mutant clones may result from the associated fate decisions that neighboring stem cells exhibit. The probability of field cancerization can be increased by the larger patches of mutant cells that can arise from the coordinated action of nearby stem cells. This process supports lobe-wide field cancerization. Although these pathways help mutant cells proliferate, they have a key drawback: since both normal and mutant cells can be lost due to continual turnover and remodeling, the same processes that enable the growth of mutant clones also aid in the eradication of many mutant cells.

Thus, it is speculated that as the number of ductal remodeling events increases, there is an increased risk of the development of a cancerized field. This is supported by earlier ages of menarche being associated with a higher risk of BC; a 2012 study showed that with each consecutive lower year of menarche, BC risk increased by a factor of 1.050 [74]. It was also found that risk increased by 1.029 for each increased age at menopause.

Because breast tissue grows and recedes in regular cycles, mutations may accumulate over time, leading to a correlation between increasing age and recurrence risk. MaSC cells and their offspring are particularly affected and may undergo oncogenic driver mutations as a lineage due to several rounds of division and exposure to various biological stressors over the course of multiple menstrual cycles. However, women under the age of 40 experience an increased risk for ipsilateral recurrence with a hazard ratio of 1.89 [75]. As the average range for menopause is 45–55 years, the increased risk for younger women supports the idea that ductal remodeling affects recurrence risk, because postmenopausal women do not undergo remodeling. However, these effects can also be attributed to an increased length of exposure to estrogen and progesterone, which are known to exhibit some carcinogenic effects over large timescales [76]. Indeed, both aberrant and wild-type cell proliferation can be encouraged by the variations in progesterone and estrogen that occur during each menstrual cycle. These hormones function as potent growth and division promoters in breast tissue. Although this is a typical aspect of the menstrual cycle, it also implies that the aberrant cells that exist could potentially multiply further under the influence of these hormones.

Nulliparity—the state of having never given birth—is also a factor for increased risk and further supports the theory that recurrence risk is related to ductal remodeling rather than estrogen exposure. Nulliparity can increase the risk for DCIS development with an odds ratio (OR) of 2.2, and the age of birth of the first child at 30 years or older can increase risk by an OR of 2.3 [77]. During pregnancy, ductal remodeling significantly differs from the monthly process during menstruation, which suggests that an increased number of pregnancies may lead to decreased risk for a mutated field. Compared with nulliparous women, an increasing number of pregnancies was associated with decreased risk at *p* trend < 0.01 [78]. Women with four or more pregnancies had a 31% lower risk. Parous women with their first full-term pregnancy at 35 years or older had an 118% greater risk than women whose first pregnancy occurred before the age of 21. Concordantly, in an article from Merill et al. that explored BC incidence in Utah, LDS women—a population with average increased parity—had an age-adjusted incidence rate of 107.6 compared to 130.5 in non-LDS women [79]. Additionally, in support of the role of ductal remodeling, parity was associated with lowered risk for ER+ BC in a 2012 study despite increased levels of estrogen during pregnancy [74].

However, although reduced BC risk is associated with higher parity, lactation—another adaptation of the monthly ductal remodeling cycle—promotes changes in the mammary microenvironment that increase risk for tumorigenesis. Lactation-induced involution can activate inflammatory pathways seen in wound-healing processes and promote tumor-related immune cell processes like angiogenesis and cellular motility [80]. Increased matrix metalloproteinase (MMP) activity also occurs during involution, and while these enzymes are important for normal processes like tissue remodeling and wound healing, they are also involved in cancer development and metastasis [81]. Involution may thus create a cancer-primed ductal microenvironment that remains conducive to tumor development. This process could explain the increased mortality of pregnancy-associated BC (PABC) [82]. However, this increased mortality can also be explained by the frequent delayed diagnosis of PABC.

#### 4.2.3. Going Viral: Viral Mechanisms Implicated in Field Cancerization

Persistent viral infections have been implicated in field cancerization via multiple mechanisms: chronic inflammation, DNA damage, and direct oncogenic effects. Combined with the fact that some viruses infect the majority of the world’s population, viral field cancerization becomes a topic of great interest. Some particularly well-documented viruses with oncogenic potential include Human Papillomavirus (HPV), Epstein–Barr Virus (EBV), Human Herpesvirus 5 (HHV-5, also called HCMV), and Hepatitis B and C viruses. Among viruses with reported oncogenic associations, EBV and HHV-5 have been investigated in BC and other epithelial malignancies. However, it must be emphasized that direct clinical evidence linking these viruses to DCIS-specific recurrence has not yet been established in human trials, and the causal relationship remains largely hypothetical and underexplored in this context [83].

#### 4.2.4. Inflammation, Immune Evasion, and Immune Suppression

**Figure 1:**
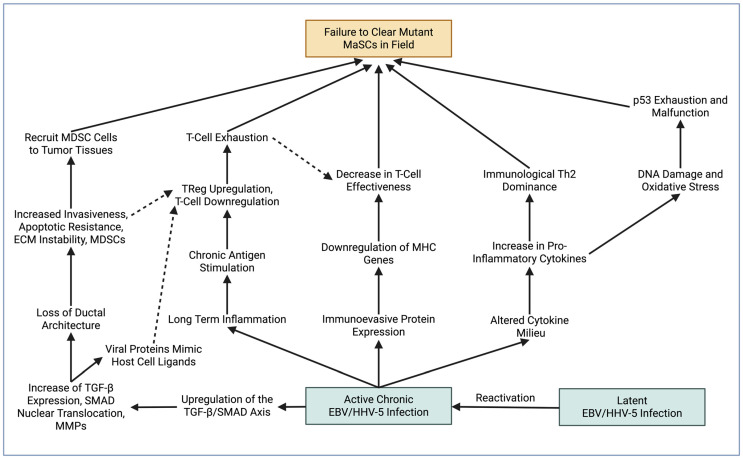
Chronic and latent viral infections as drivers of field cancerization through immune dysregulation and inflammatory signaling. Solid arrows indicate direct regulation, while dotted arrows indicate indirect or predicted regulation.

The immune system plays an important role in maintaining mammary tissue homeostasis by identifying and eliminating aberrant cells, especially those with oncogenic potential. Persistent infection with oncogenic viruses such as EBV and HHV-5 has been shown in multiple malignancies to alter immune surveillance, disrupt tumor suppressor pathways, and reprogram host signaling networks (Figure 1) [84,85,86,87,88,89]. In established cancers, these viruses can downregulate antigen presentation, promote T-cell exhaustion, skew cytokine milieus toward immunosuppressive states, and interfere with key regulatory nodes including p53 and RB [86,87,88,89,90,91,92]. Chronic inflammatory signaling induced by viral persistence has also been linked to oxidative stress and accumulation of DNA damage in epithelial tissues [90,91]. Within the context of the breast ductal system, such immune dysregulation could plausibly impair clearance of aberrant mammary stem or progenitor cells during cyclical remodeling, permitting mutant clones to persist and expand across a wider epithelial field.

Beyond immune escape, viral gene products have been reported to modulate pathways such as TGF-β/SMAD, PI3K/AKT, MAPK, and mTOR in diverse tumor models [93,94,95,96,97,98,99,100,101,102,103,104,105,106,107]. TGF-β signaling in particular exhibits a dual role in carcinogenesis, acting as a tumor suppressor in early epithelial contexts while promoting invasion, immune suppression, and epithelial–mesenchymal transition (EMT) in later stages [93,99]. Viral perturbation of this axis may therefore shift the balance toward pro-tumorigenic outcomes. EMT-associated programs not only enhance motility and apoptotic resistance but may also confer stem-like phenotypes, increasing the likelihood that altered ductal epithelial cells clonally propagate within a susceptible field.

Viral-driven signaling may further influence the stromal compartment. Activation of TGF-β-dependent pathways and induction of MMPs have been associated with extracellular matrix remodeling, fibroblast activation, and collagen deposition in several malignancies [108,109,110,111]. Resultant increases in tissue stiffness and altered mechanical signaling have been linked to enhanced epithelial proliferation and invasive behavior [112,113]. Such stromal remodeling could reinforce the survival and expansion of genetically or epigenetically altered clones beyond the primary lesion in a field cancerization context.

Reactivation of latent viral reservoirs under conditions of inflammation, hypoxia, or immune suppression may intermittently amplify these processes through episodic expression of immediate-early or latency-associated proteins that perturb cell cycle control and epigenetic regulation [114,115,116,117,118,119,120,121,122,123]. Conceptually, this raises the possibility that latent infection could sustain or periodically intensify pro-survival and immunosuppressive signaling within tumor-adjacent tissues. However, whether latent viral reservoirs within breast tissue harbor premalignant clones or directly contribute to multifocality or ipsilateral recurrence remains unproven.

Importantly, the majority of mechanistic data supporting these paradigms derive from invasive cancers or non-breast malignancies. Direct clinical evidence linking EBV or HHV-5 infection to DCIS-specific ipsilateral recurrence or ductal field cancerization remains limited. At present, viral contributions to recurrence risk in DCIS should therefore be regarded as mechanistically plausible but speculative. If operative, viral influences would most likely function as amplifiers of pre-existing genomic instability or immune dysfunction within a susceptible ductal field, rather than as primary initiating events. Rigorous studies quantifying viral burden, latency markers, immune exhaustion signatures, and stromal remodeling patterns in DCIS tumor-adjacent tissues will be required to determine whether these mechanisms meaningfully extend to DCIS recurrence biology.

This schematic illustrates how persistent or reactivated viral infections, such as Epstein–Barr virus (EBV) or Human Herpesvirus-5 (HHV-5), may contribute to field cancerization by impairing immune-mediated clearance of aberrant mammary stem and progenitor cells. Failure to eliminate these mutant cells creates a permissive niche in which latent viruses may reactivate, promoting chronic antigenic stimulation, long-term inflammation, and progressive immune exhaustion. Viral proteins mimic host ligands and interfere with antigen presentation through downregulation of MHC genes, leading to reduced T-cell effectiveness, regulatory T-cell expansion, and recruitment of myeloid-derived suppressor cells (MDSCs). Concurrently, altered cytokine milieus—specifically those marked by increased pro-inflammatory and Th2-skewing signals—drive oxidative stress, DNA damage, p53 dysfunction, and epigenetic instability. Viral activation of pathways such as TGF-β/SMAD and MMP expression further disrupts ductal architecture, enhances extracellular matrix remodeling, and promotes apoptotic resistance. Together, these interconnected viral, immune, and stromal alterations establish a pro-tumorigenic field that facilitates survival, expansion, and malignant progression of genetically altered cells. Figure created using BioRender.

## 5. Post-Tumorigenesis: Secondary Drivers of Field Cancerization

Local recurrence was once thought to only result from the contamination of the cells in the surgical margin by tumor cells or by undetected escapee tumor cells from the primary DCIS tumor [124]. However, a vast collection of studies showing that peri-tumoral cells harbor distinct genetic, epigenetic, transcriptomic, and telomeric aberrations has challenged this paradigm and instead suggest that the milieu of the tumor margin predisposes cells towards accruing pro-tumorigenic alterations and undergoing neoplastic transformation [125,126,127,128,129,130,131,132,133,134,135,136]. Furthermore, in the early stages of cancer-priming molecular alterations, cells may not show stark or obvious changes in tissue phenotype and may appear “histologically normal.” These studies also support the idea that peri-tumoral tissues may possess distinct and prognostically significant information that may augment traditional histopathological examination to enable more accurate risk stratification of patients and better BC management [126,127,137,138].

## 6. The Crux of the Matter: Genomic Instability Is Widespread in Peritumoral Tissues

Genomic instability, which refers to the increased frequency of genomic changes and mutations that contribute to dysregulation of cellular proliferation and/or tumor suppression pathways, typifies most cancers. Genomic instability allows cancer cells to sample a variety of genomic states, and any state that enhances the survival of tumor cells may be selected for and propagated to progeny cells. Allelic imbalance (AI), a deviation from the normal 1:1 ratio of alleles, and loss of heterozygosity (LOH), the complete loss of an allele, are two common manifestations of genomic instability. More than 150 published analyses of breast tumors have found frequent allelic imbalance in several chromosomal regions. Multiple studies have shown an accumulation of these Ais during tumor development, such that invasive lesions often show an AI frequency of 25–50% [139]. In tumor-adjacent histologically normal breast tissue, both AI and LOH are commonly found. Being distance-dependent, high rates of LOH are found very close to the tumor, and higher rates of AI are within the 1 cm margin of the tumor [140].

These genetic abnormalities can exist pre-tumorigenesis. It was reported that 22% of matched normal breast tissue adjacent to reduction mammoplasty, atypical hyperplasia, and adenocarcinoma tissue exhibited genetic abnormalities [141]. This finding was further supported by a follow-up study that yielded similar results after analyzing LOH in histologically normal tumor-adjacent tissue [142]. At a 5 cm tumor distance, the extent of AI was similar to the extent of AI in reduction mammoplasty tissue. However, it was also observed that at a 1 cm tumor distance, there was a 5-fold increase in the incidence of AI in histologically normal tissue. These observations suggest that a field of cells surrounding the tumor (of at least 1 cm) are consitent with the presence of genetic abnormalities, likely influenced by the adjacent tumor’s presence. It has also been reported that a specific LOH, at chromosome 3p11-26 in histologically normal breast tissue, correlates with local recurrence (HR: 3.9–5.2), even after adjusting for confounding variables, such as tumor grade and marginal tumor distance [143]. Whole genome analysis of breast tumor and adjacent histologically normal tissues revealed that the most common alteration was gain of an ERBB2 gene copy, ERBB2 genomic amplification, and amplification of other growth factor receptor genes (i.e., EGFR, FGFR1, IGF1R, LIFR, and NGFR) [131].

The drivers of increased genomic instability in histologically normal tumor-adjacent tissues or tumor-adjacent stroma are largely unknown. However, it is known that genomic instability can develop in response to both endogenous and exogenous factors. Some of these factors include lifetime estrogen exposure, which has been discussed previously in this review [76]. Estrogen metabolites can drive the development of HR+ breast tumors by increasing cellular proliferation via estrogen-receptor-mediated gene transcription or by destabilization of DNA through depurination [144].

These genomic alterations can support a pro-tumor microenvironment and predispose pre-malignant tissue to undergo tumorigenesis or increase risk for local recurrence. The frequency of AI in adjacent tissue (15.4%) is substantially higher than in distant tissue (3.7%) [145]. This observation suggests that independent genomic changes in morphologically healthy breast tissues are not sporadic genetic anomalies because they occur with appreciable frequency, and that genomic instability might be inherently greater in tissue adjacent to invasive and in situ carcinomas. Klimov et al. leveraged machine learning to build a classifier that predicted the 10-year risk of DCIS ipsilateral recurrence risk using digitized whole slide imaging of H&E-stained DCIS sections. In their study, tumor sections were extracted post-BCS along with accompanying clinicopathologic long-term outcome data. It turned out that 6 out of 8 sub-visual features used in the final classifier were extracted from the tumor microenvironment and the margins of the tumor. This finding suggested that there is a wealth of unharnessed prognostically relevant information present in the TME as well as in the histologically normal peritumoral regions that is subvisual [138]. Further, it has been suggested that spatial features within the margin, such as increased blood vessel and micro vessel density or immune-rich stroma, have prognostic value that is often overlooked. One study showed that among 355 DCIS cases, 32 developed recurrent disease. In the matched controls of these 32 recurrent cases, periductal vascular density was quantified within a 2 mm margin, and this feature was found to be associated with risk of invasive and in situ recurrence. Furthermore, micro-vessel density was found to be greater around DCIS foci compared to histologically normal breast lobules and was significantly higher in cases of DCIS that recurred [146]. Collectively, these alterations in the tumor margin, including increased blood vessel density, microvessel density, and malignant characteristics, may reflect a cancerized field. These observations also demonstrate the tumor’s influence on both histologically normal cells within the tumor margin and on the tumor microenvironment, and highlight the importance of studying the properties of tumor-adjacent histologically normal breast tissue, irrespective of physical distance, to discern the extent of post-tumorigenesis field cancerization. Prospective studies leveraging artificial intelligence and designing machine learning-based classifiers that successfully integrate and harness prognostic value from the surrounding tumor stroma and margin regions to refine patient risk-stratification may significantly improve and better guide DCIS patient management than current lesion-focused practices and classifiers.

One major driver that has been suggested to underlie elevated levels of genomic instability in tumor-adjacent histologically normal breast tissue is centrosome amplification (CA)—a hallmark of malignancy—wherein cells harbor an above-average number of centrosomes and/or centrosomes with abnormally high volumes. CA engenders chromosomal instability and intratumoral heterogeneity, which has been suggested to promote an aggressive disease course in several types of solid tumors [19]. Since centrosomes organize the microtubules for proper cell division, supernumerary centrosomes coalesce into opposite sides of the cell via centrosome clustering to circumvent a catastrophic multipolar spindle mitosis. The transient multipolar state that exists just prior to centrosome clustering leads to a low and tolerable level of whole chromosome mis-segregation. AI is one of the most common outcomes of CA. It has been reported that DCIS cases with local recurrences exhibit significantly greater levels of CA than recurrence-free DCIS cases [147]. High CA was linked to a greater 10-year risk of local recurrence and CA was determined to be an independent predictor of relapse-free survival [148]. Importantly, the study by Mittal et al. [149] found that tumor-adjacent histologically normal cells showed extensive CA, suggesting that cells in the peri-tumoral margin showed deregulation of the centrosome duplication cycle. Since CA is considered to be one of the earliest readouts of cellular transformation, these findings suggest that peri-tumoral margins have undergone alterations potentially indicative of cancerization. Denu Ra et al. demonstrated that CA in non-transformed human cells induces a cellular state and features similar to high-grade malignancy [150]. These findings collectively suggest that high levels of CA present in tumor-adjacent histologically normal breast tissue likely reflect a field exhibiting pre-malignant characteristics. Furthermore, this field can harbor valuable risk-prognostic information. Specifically, quantifying the prevalence and severity of CA in histologically normal tumor-adjacent tissue may be critical to understanding the tumor’s influence on surrounding mammary tissue and risk of local recurrence. We previously developed and reported a semiautomated methodology that integrates immunofluorescence confocal microscopy and digital image analysis to quantitate the severity and frequency of numerical and structural CA in DCIS lesions to assign each patient a centrosome amplification score (CAS) [149]. Within the analyzed cohort, CAS was found to predict local recurrence and to be an independent predictor of relapse-free survival, even after adjusting for confounding variables such as age, grade, and radiotherapy. Further, CAS was able to better risk-stratify DCIS patients for local recurrence than the Van Nuys Prognostic Index. However, immunohistochemistry (IHC), in lieu of immunofluorescence confocal microscopy, is the preferred routine clinical methodology to risk-stratify patients, deeming CAS not feasible for routine pathology. Also, IHC cannot feasibly calculate centrosome volume to adequately capture structural amplification in patient tissue specimens, further compounding the challenge of translating CAS determination for risk-prediction in the clinic.

Another possible mechanism of genomic instability driving field cancerization is mutations in the BRCA1 and BRCA2 tumor suppressor genes. BRCA1 and BRCA2 mutations are responsible for the vast majority of breast and ovarian cancer development. These genetic mutations, when inherited or produced sporadically, are known to alter mammary epithelia to increase susceptibility to tumorigenesis [151]. Both genetic mutations play a critical role in the S phase of the cell cycle. Further, BRCA1 is involved in transcriptional regulation of both p53-dependent and independent responses, while BRCA2 is involved in homology-directed repair by interacting with Rad51 and directly binding to single-stranded DNA [130]. Thus, BRCA1 and BRCA2 mutations could be a major driver of increased frequency of AI and LOH in tumor-adjacent tissue. In addition, in cases wherein these mutations are present in all cells of the body, the entire body can potentially be viewed as a cancerized field—a broad, genomically unstable landscape in which multiple independent clones have an elevated likelihood of initiating and progressing to cancer.

The genomic, stromal, and microenvironmental alterations described above support the development of margin-focused molecular assays that complement existing lesion-centric recurrence tools. Quantifiable biomarkers within histologically normal peri-tumoral tissue could include: (1) locus-specific loss of heterozygosity or allelic imbalance at recurrence-associated regions such as 3p11-26; (2) ERBB2 copy number gain or amplification in adjacent morphologically normal epithelium detected by in situ hybridization; (3) immunohistochemistry-adapted centrosome amplification metrics as a surrogate for chromosomal instability; (4) standardized microvessel or periductal vascular density within a defined 2 mm margin; and (5) composite genomic instability indices integrating copy number alterations across margin tissue. Incorporating these measurable field biomarkers into existing nomograms or gene-expression based platforms may refine recurrence prediction beyond margin width alone. Prospective validation of margin-derived biomarkers in independent cohorts would be required before clinical implementation.

## 7. Top-Down Effect: Epigenetic and Transcriptomic Changes in Tumor Margin Cells

Comparison of the methylation landscape between tumor cells and histologically normal cells from matched normal tumor-adjacent regions has shown that the cells from the margins of a tumor are epigenetically distinct from cells extracted from contralateral breast tissue [129]. Promoter hypermethylation has been observed both in in situ breast carcinomas as well as in tumor-adjacent tissues, and its presence in tumor-adjacent tissues has also been proposed to potentially underlie local recurrence [152,153,154,155]. In tumor-adjacent tissues, promoter hypermethylation has been observed in genes such as RARb, RASSF1A, and APC [126,128]. Epigenetic alterations in histologically normal peri-tumoral cells have a significant impact on gene expression. Transcriptomic studies have uncovered that the frequency and severity of transcriptional dysregulation in peri-tumoral cells depends on the distance from the primary tumor—a trend similar to that observed with genomic instability [156,157,158]. Transcriptomic analyses of tumor-adjacent tissues show that these tissues have gene expression profiles that are distinct from those of the adjacent tumor but also exhibit certain features that are quite similar to those of the tumor, such as extensive deregulation of pathways involved in extracellular matrix remodeling, EMT, and altered metabolism. Alterations in cell signaling within the 1 cm margin, including loss of cell–cell junction proteins—such as E-cadherin, and a gain and re-arrangement of cytoskeletal proteins such as α-smooth muscle actin (α-SMA) and vimentin—lead to a loss of epithelial cell characteristics and the adoption of mesenchymal cell properties. Driven by transcription factors such as SNAIL and TWIST, EMT also results in the generation of stem cell populations [135]. Prognostic signatures have been developed from gene expression data derived from tumor-adjacent tissues [134,137,159,160,161]. Collectively, these findings suggest a genetic and epigenetic influence of the tumor on tumor margin cells, which alters the tumor’s surroundings to facilitate tumor progression and likely local recurrence. In support of this hypothesis, a study that analyzed TCGA data found that the gene expression profiles of peri-tumoral tissues were stronger predictors of patient survival than gene expression profiles of tumor tissues [162].

MicroRNAs (miRNAs) are a key epigenetic regulator of protein-coding genes. They function as both targets of epigenetic changes and as regulators of epigenetic modifiers. By binding to the mRNAs’ 3′ UTRs, miRNAs destabilize and translationally repress target mRNAs [163]. Although we lack knowledge on direct epigenetic changes in the margins of breast tumors, the heightened presence of miRNAs is an important biomarker in triple-negative BC [159,164]. As exosomes transfer miRNAs and other small cargo molecules, there is a strong possibility of the transfer of epigenetic modulators into cells surrounding the tumor margins—a process that warrants further investigation [165].

### 7.1. Shifting Gears: Telomere Erosion and Telomerase Expression in Tumor-Adjacent Tissues

Genetic abnormalities have also been discovered in the telomeres within tumor-adjacent histologically normal breast tissue. Within the 1 cm margin of the tumor, telomeres have been found to be 30–40% shorter on average than in paired peri-tumoral tissues that are at a 5 cm marginal distance [166]. Telomerase expression has also been observed in tumor = adjacent histologically normal breast tissue within the 1 cm margin of the tumor, which suggests the presence of a cell population on a path towards immortalization.

### 7.2. The Bystander Effect: Other Changes Observed in Peritumoral Tissues

Studies have also demonstrated that gut microbial dysbiosis triggered by antibiotic regimens is associated with increased breast tumor metastasis in murine models [167]. Additional studies have shown that a connection may exist between the health of the gut microbiome and the mammary microbiome, and this link may impact disease course. Distinct microbial signatures are associated with different BC subtypes, and the microbial profile of tumor-adjacent tissues is more similar to that of the associated tumor than to that of healthy controls. Thus, the gut and breast microbiomes could potentially be manipulated to circumvent BC development or improve therapeutic outcomes for BC patients [168,169,170,171]. It is presently unclear whether mammary microbiome dysbiosis alters the tumor microenvironment or if field cancerization fosters dysbiosis.

### 7.3. Home Invasion: Roles of Exosomes in Field Cancerization

Exosomes are small extracellular vesicles that play a critical role in intercellular communication. While previously thought to be cellular waste, exosomes are now known to carry bioactive molecules such as proteins, lipids, and RNAs, which can influence the behavior of the adjacent field. Exosomes are thus gaining recognition as key players in tumor progression, immune modulation, metastasis, and drug resistance, and may also serve as potential diagnostic and therapeutic tools [172]. Accumulating studies are beginning to suggest that cancer cells release significantly more exosomes than normal cells, even during the earliest stages of cancer development [173].

Exosomes primarily contribute to cancer progression by modulating the tumor microenvironment. Tumor-derived exosomes influence surrounding stromal and immune cells to promote angiogenesis, immune evasion, and extracellular matrix remodeling [174,175]. Notably, exosomes can carry integrins and specific RNA or protein cargo that determine organotropic metastasis patterns, possibly enhancing metastatic efficiency [176]. Collectively, these observations support a model in which tumor-derived exosomes act as information-rich signals that can extend malignant influence well beyond the primary lesion, “conditioning” adjacent tissue to create a tumor-permissive microenvironment among otherwise histologically normal cells.

## 8. Exosomal miRNAs

Many miRNAs have the potential to act as oncogenes and may contribute to changes in the TME. For example, miR-9 and miR-200s have been found to convert normal fibroblasts into cancer-associated fibroblasts (CAFs) and enhance metastasis; miR-526b and miR-655 may facilitate blood and lymph vessel formation, and miR-340-5p and miR-561 may help to build an immunosuppressive environment [177,178]. Understanding how these miRNAs are delivered and function in both tumor cells and the TME is a key area of research.

Exosomes derived from tumor cells have been demonstrated to play a critical role in amplifying cancer invasiveness by transporting specific miRNAs. IL-4 prompts macrophages to secrete exosomes carrying oncogenic miRNA-223, which targets the Mef2c-β-catenin pathway in BC cells [179]. This interaction increases nuclear β-catenin levels to promote cell migration and invasion. Exosomes can also transport miRNAs such as miRNA-105 from the primary tumor to distant sites, where they disrupt tight junction proteins in endothelial cells, increase vascular permeability, and rapidly increase the process of metastasis [180].

Furthermore, exosomes may act as messengers within the tumor and within the margin or distant tissues to activate tumor cells, endothelial cells, and fibroblasts that aid metastatic growth [181]. In esophageal squamous cell carcinoma (ESCC), researchers discovered elevated levels of miRNAs, particularly miRNA-21, in serum exosomes of affected individuals [182]. These exosomes promoted ESCC cell proliferation in vitro. suggesting that this disease-specific miRNA packaging into exosomes drives this proliferative activity.

Tumor-derived exosomes may also influence gene expression and signaling in vascular endothelial cells by transferring miRNAs and tissue factors that promote angiogenesis and tumor aggression [183]. Such intercellular communication may extend pro-tumorigenic signaling beyond the primary lesion and build a tumor-permissive microenvironment in histologically normal adjacent tissue.

### 8.1. Exosomal miRNA Within Stromal Cells

Exosomes have been shown to act as critical messengers that facilitate tumor-stroma communication. Yan et al. revealed that BC cells can secrete extracellular vesicles rich in miR-105 to reprogram neighboring CAFs, which extends tumor-promoting metabolic effects beyond cancer cells themselves [183]. When uptaken by CAFs, miR-105 targets MXI1, a MYC inhibitor, thereby enhancing MYC signaling and triggering the upregulation of genes involved in glycolysis, glutaminolysis and metabolite transport, such as *HK2*, *LDHA*, *GLS*, and *SLC2A1* [184]. These alterations raise levels of lactate and glutamine metabolism in fibroblasts, which may allow them to support tumor cells under metabolic stress. Notably, this exosomal miR-105-driven reprogramming alters the extracellular environment. In high-lactate conditions, CAFs enhance bidirectional lactate-pyruvate conversion and secrete lactate-derived acetate and glutamate, mitigating acidosis and supplying alternative fuels to cancer cells. Similarly, in glutamine-depleted, ammonium-rich environments, these CAFs can convert NH_4_^+^ into glutamate, glutamine, and nucleotides like UMP—nutrients essential for biosynthesis and redox balance in proliferating tumor cells. This activity detoxifies the tissue microenvironment and allows for cancer cell survival, growth, and eventual migration [185].

In murine models where BC cells expressing high levels of miR-105 were transplanted, elevated levels of labeled metabolic intermediates were exhibited, along with enhanced cancer cell proliferation. Research also demonstrates that blocking (MCTs), which mediate acetate and lactate exchange between CAFs and tumor cells, may reverse these effects, supporting the role of metabolic coupling in tumor progression. Thus, exosomes can act as systemic modulators, reprogramming the stromal cells to establish a nutrient-rich, detoxified environment that can support tumor expansion well beyond the original tumor borders [153].

### 8.2. Exosomal Cytokines, MMPs and Hypoxia-Inducible Factor, and Other Proteins

**Figure 2:**
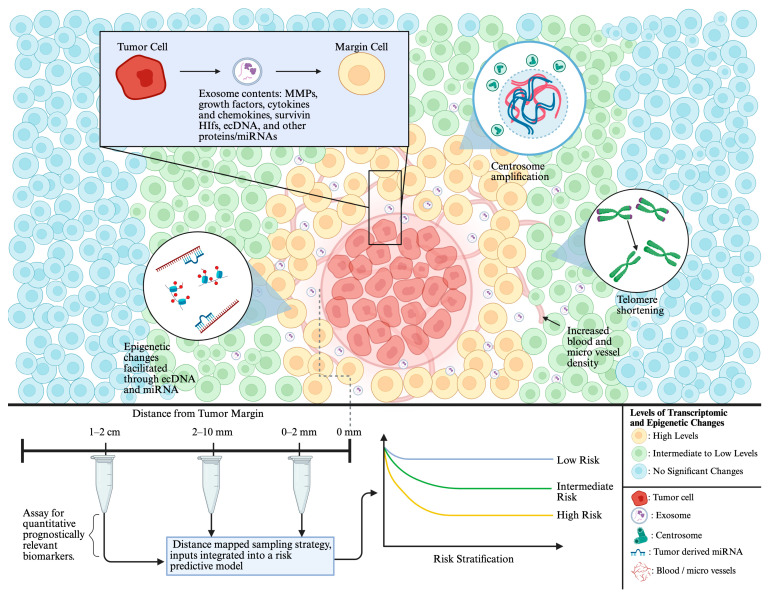
The top panel of this figure depicts biologically plausible mechanisms by which established tumor cells remodel adjacent, histologically normal tissue to extend a pro-tumorigenic field beyond the visible tumor boundary. Tumor cells release extracellular vesicles (exosomes) enriched in MMPs, growth factors, cytokines and chemokines, survivin, hypoxia-inducible factors (HIFs), extrachromosomal DNA (ecDNA), and regulatory RNAs (mRNAs and miRNAs), which are taken up by neighboring margin cells. This intercellular transfer may induce epigenetic reprogramming, alter transcriptional and translational states, and promote centrosome amplification, telomere shortening, and genomic instability in margin cells. Concurrently, tumor-derived signals drive extracellular matrix remodeling, angiogenesis, immune modulation, and survival of stem-like cell populations, facilitating EMT and resistance to apoptosis. Together, these processes may convert surrounding tissue into a tumor-affected, biologically altered margin that remains permissive for local recurrence despite appearing histologically normal. The bottom panel proposes a distance-based sampling framework as a translational bridge to risk stratification. The pro-tumorigenic gradient is illustrated relative to the resection boundary (tumor bed, 0–2 mm, 2–10 mm, 1–2 cm). Biomarker data derived from spatially defined samples—including AI, telomere attrition, epigenetic alterations, proliferation and CIN programs, immune and stromal remodeling signatures, and vascular context—may be integrated with lesion-centered biomarkers and prognostic variables into a multivariable model to stratify patients based on potential recurrence risk, described later. The figure highlights how field-informed risk states can potentially complement lesion-centric pathology to guide individualized clinical decisions regarding re-excision extent, radiotherapy benefit, surveillance intensity, and safe de-escalation in patients with biologically low-risk fields. Figure created using BioRender.

Exosomes can also serve as key transporters of proteins that significantly influence the TME and adjacent tissues. Among these, hypoxia-inducible factors (HIFs), MMPs, and inflammatory cytokines have essential roles for shaping the TME and promoting adjacent-tumor progression (see Figure 2) [154,155]. Hypoxia, a common trait in rapidly growing tumors, triggers the stabilization of HIF-1α and HIF-2α. These HIFs activate the transcription of genes critical for survival under low oxygen conditions. One of the most significant downstream effects of HIF activation is the upregulation of vascular endothelial growth factor (VEGF), which is a key promoter of angiogenesis [186]. This increased circulatory access allows sustained tumor growth and metastasis in surrounding tissues. Hypoxia increases exosome release from BC cells in a HIF-1α-dependent manner. These exosomes may carry enhanced levels of pro-angiogenic and pro-invasive proteins, causing exosome-mediated remodeling of the TME and adjacent tissues [155]. In support, recent studies have shown that HIF-1α is not only activated within tumor cells but can also be packaged into exosomes and delivered to surrounding stromal cells, including CAFs and endothelial cells [176]. This transfer reinforces hypoxia-driven signaling in neighboring cells, amplifies the angiogenic response, and reinforces TME heterogeneity.

Exosomes can also carry MMPs—particularly MMP2 and MMP9—which degrade ECM components, facilitating tumor invasion. In hepatocellular carcinoma (HCC), exosomal miR-21 upregulates MMP2 and MMP9 via activation of the PDK1/Akt signaling pathway in CAFs, promoting angiogenesis and ECM remodeling [155]. Exosomal delivery of MMPs or the miRNAs that regulate MMPs may directly contribute to breaking down tissue barriers, enabling cancer cells to migrate and invade adjacent sites. Moreover, exosome-mediated distribution of MMPs can enhance the dynamic remodeling of the ECM, not just around the malignant cells but throughout the TME, fostering a more permissive environment for tumor progression [186].

In parallel, exosomes can carry inflammatory cytokines or induce their production in target cells. Cytokines such as IL-6, IL-8, and TGF-β are frequently upregulated in the TME and are critical for establishing a chronic inflammatory state that supports tumor development [176]. For example, exosomal miR-1247 from tumor cells targets the B4GALT3 gene in fibroblasts, which activates the β1-integrin–NF-κB pathway, resulting in the secretion of IL-6 and IL-8. These cytokines not only promote inflammation but also enhance angiogenesis, immune cell recruitment, and tumor cell survival [187]. CAFs, once activated, adopt a highly secretory phenotype characterized by the continuous release of cytokines, chemokines, and growth factors, many of which further stimulate tumor growth and metastasis. Thus, exchange of exosome-mediated signals between breast tumor cells and histologically normal cells in the margin may serve as a post-tumorigenic mechanism for field cancerization. By transferring oncogenic proteins, miRNAs, cytokines, and potentially even ecDNA into adjacent tissues, tumor exosomes can conceivably reprogram the surrounding field to be predisposed to tumorigenesis.

PCSK9 is a secreted protein primarily known for its role in regulating low-density lipoprotein receptor (LDLR) levels and cholesterol homeostasis. However, a recent study identified a germline missense variant in PCSK9 (rs562556, V474I) that is associated with increased metastatic relapse and reduced survival in BC patients [188]. Using genetic modeling of the PCSK9 rs562556 variant in mice, the study demonstrated that this gain-of-function mutation promotes BC metastasis. Deletion of PCSK9 reduced metastatic colonization in multiple BC models, further supporting that PCSK9 activity is vital in driving metastasis. Mei et al. showed that PCSK9 enhances metastasis by targeting LRP1 on tumor cells, suppressing genes that repress metastasis and promoting the survival of these cells. PCSK9 also appears to influence exosome biogenesis and cargo loading. Elevated PCSK9 levels were associated with increased secretion of exosomes enriched with pro-metastatic molecules, including integrins and growth factors. These exosomes can be transferred to histologically normal cells in the tumor margin, potentially predisposing these cells to tumorigenesis. Concurrently, Tan et al. found enhanced metastatic initiation and colonization in distant organs, providing a direct link between PCSK9 activity and exosome-mediated metastatic signaling.

### 8.3. Exosomal Growth Factors

Growth factors in the TME function as potent modulators of cellular behavior via complex autocrine and paracrine signaling. Rather than acting in isolation, these molecules operate in tightly regulated—and often dysregulated—feedback loops that enable tumor cells to communicate with surrounding stromal, immune, and endothelial cells [189]. Tumor-derived exosomes may distribute growth factors and regulatory RNAs across the TME and over into cells in the tumor margin, allowing tumor cells to reprogram neighboring cells, and potentially predisposing the wider margin to recurrence. One of the key features of growth factor signaling in the TME is its self-sustaining nature. Tumor cells not only respond to growth factors like VEGF, FGF2, and TGF-β, but also produce and secrete them in exosome-associated forms, creating continuous activation signals [162]. This feedback signaling loop is particularly evident in CAFs, which secrete additional growth factors upon tumor-derived cues that further reinforce tumor growth and metastasis. Exosomes from both tumor cells and CAFs contain growth factor mRNAs or modulatory miRNAs that enhance this effect by altering gene expression in recipient cells.

Moreover, growth factors help establish spatial organization within the TME. VEGF gradients, for instance, guide endothelial cell migration and vessel patterning in addition to promoting angiogenesis, contributing to the chaotic vasculature typical of aggressive tumors [190]. FGF and HGF contribute similarly to tissue remodeling and cellular motility. TGF-β, beyond its immunosuppressive and EMT-promoting roles, also induces fibrotic changes in the ECM, creating a stiffened microenvironment that favors invasion and can be transferred via exosomes to adjacent cells, promoting cancer cell plasticity and resistance to therapy in the field [191]. Exosomal trafficking allows these growth factor-related signals to reach not only adjacent cells but also distant pre-metastatic niches, potentially priming tissues for future colonization.

### 8.4. Exosomal Survivin

Survivin, a member of the inhibitor of apoptosis family, has also been demonstrated to have a critical role in tumor cell survival, cell division, and resistance to stress. Often overexpressed in cancerous tissues and absent in most normal adult cells, survivin contributes to cancer progression by enhancing mitotic activity and inhibiting apoptotic pathways. It exists in multiple cellular pools—including the cytoplasm, nucleus, and mitochondria—and has also been identified in extracellular vesicles, particularly exosomes. Exosomal survivin is of particular interest due to its ability to influence the TME by transferring its pro-survival and stress-resistance properties to adjacent cells, supported by a 2011 study that studied cervical carcinoma cells [192]. This exosomal dissemination can enhance tumor cell invasiveness, resistance to chemotherapy, and immune evasion. The loading of survivin into exosomes is often upregulated under stress conditions such as chemotherapy or radiation, potentially through p53-regulated pathways like TSAP6. A later study from the same lab found survivin and survivin splice variants in exosomes in breast cancer patients’ sera, supporting the idea that survivin is released to adjacent cells, predisposing the field to recurrence, particularly in BC [165]. Notably, survivin has been proposed as a non-invasive poor-prognosis biomarker due to its presence in circulating exosomes found in patient plasma and other body fluids.

### 8.5. ecDNA as a Driver of Breast Tumor Metastasis

Extrachromosomal DNA (ecDNA)—large, circular DNA fragments that often carry oncogenes and are strongly associated with genomic instability and aggressive cancer phenotypes—can arise from genomic instability, especially from chromothripsis, which refers to massive chromosomal rearrangement due to DNA damage. Although there is presently no direct evidence of ecDNA being present in tumor-adjacent histologically normal breast tissue, ecDNA most commonly encodes oncogenes, including EGFR, MYC, CDK4, and MDM2. These oncogenes are among the top 1% of genes expressed in malignant cells [193]. ecDNA numbers vary significantly from cell to cell within cultured cancer cells. Gene amplification enables an oncogene to generate a large number of copies [194]. Since ecDNAs lack a centromere, ecDNAs cannot be organized by the mitotic spindle and, therefore, tend to be segregated unequally between daughter cells, which leads to intratumoral genetic and phenotypic heterogeneity [195]. It has been demonstrated that when ecDNA carries amplified oncogenes like *MYC* and *EGFR*, it leads to elevated gene expression that fuels tumor growth and adaptability [196]. In metastatic BC, ecDNA enhances cellular plasticity by dynamically adjusting oncogene dosage in response to stress, such as chemotherapy or immune pressure. This adaptability allows cancer cells to survive, invade distant organs, and resist treatment. Recent studies also suggest a potential interaction between ecDNA and exosomes. Tumor cells may load ecDNA fragments or transcripts into exosomes, promoting the horizontal transfer of oncogenic material to adjacent cells [189]. This process could contribute to increased tumor heterogeneity and metastatic propensity. These findings position ecDNA as not only a potential marker of aggressive disease but also suggest its role in establishing a field of altered cells predisposed to tumorigenesis.

### 8.6. Immune Modulation by Tumor Cells

Immune modulation within and adjacent to the tumor margin is crucial to shaping the TME and establishing a pro-tumorigenic field. On the immune-activating side, tumor-derived exosomes can trigger antitumor responses. For example, DNA-rich exosomes from LATS1/2-deficient tumor cells activate the TLR-IFN pathway, enhancing innate immunity (Moroishi et al.) [197]. Similarly, DNA from topotecan-treated cancer cells activates dendritic cells (DCs) via CGAS-STING signaling, leading to CD8+ T cell responses. Exosomes also present tumor antigens and MHC molecules, which can activate helper T cells [198]. In the same vein, tumor exosomes also promote immune suppression. They inhibit T cell and NK cell activation by delivering TGF-β, FasL, or PD-L1, which dampen cytotoxic function or induce apoptosis [199]. High levels of exosomal PD-L1 are linked to tumor burden and resistance to checkpoint inhibitors. Exosomes also impair innate immune responses; for instance, they may inhibit DC maturation, promote expansion of myeloid-derived suppressor cells (MDSCs), and reprogram macrophages toward the tumor-promoting M2 phenotype [200]. This is mediated by proteins like glycoprotein 130 or miRNAs such as miR-21 and miR-1246, which modulate STAT3 signaling and suppress M1-like immune responses. Collectively, these mechanisms indicate that immune modulation through exosomes extend beyond the primary tumor and reprogram surrounding histologically normal cells. Exosomes have been shown to dually stimulate antitumor activity and promote immune evasion to not only support the primary tumor, but also predispose the adjacent tissue to recurrence following surgical excision. In summary, tumor-margin communication may sustain and expand a cancerized field through diverse mechanisms. Margin profiling and data derived from distance-based mapping of margin biomarkers, which are depicted in Figure 2, may also be used to potentially inform risk stratification.

The top panel of this figure depicts biologically plausible mechanisms by which established tumor cells remodel adjacent, histologically normal tissue to extend a pro-tumorigenic field beyond the visible tumor boundary. Tumor cells release extracellular vesicles (exosomes) enriched in MMPs, growth factors, cytokines and chemokines, survivin, hypoxia-inducible factors (HIFs), extrachromosomal DNA (ecDNA), and regulatory RNAs (mRNAs and miRNAs), which are taken up by neighboring margin cells. This intercellular transfer may induce epigenetic reprogramming, alter transcriptional and translational states, and promote centrosome amplification, telomere shortening, and genomic instability in margin cells. Concurrently, tumor-derived signals drive extracellular matrix remodeling, angiogenesis, immune modulation, and survival of stem-like cell populations, facilitating EMT and resistance to apoptosis. Together, these processes may convert surrounding tissue into a tumor-affected, biologically altered margin that remains permissive for local recurrence despite appearing histologically normal. The bottom panel proposes a distance-based sampling framework as a translational bridge to risk stratification. The pro-tumorigenic gradient is illustrated relative to the resection boundary (tumor bed, 0–2 mm, 2–10 mm, 1–2 cm). Biomarker data derived from spatially defined samples—including AI, telomere attrition, epigenetic alterations, proliferation and CIN programs, immune and stromal remodeling signatures, and vascular context—may be integrated with lesion-centered biomarkers and prognostic variables into a multivariable model to stratify patients based on potential recurrence risk, described later. The figure highlights how field-informed risk states can potentially complement lesion-centric pathology to guide individualized clinical decisions regarding re-excision extent, radiotherapy benefit, surveillance intensity, and safe de-escalation in patients with biologically low-risk fields. Figure created using BioRender.

## 9. Connecting the Dots: Shifting Emphasis from “Clear Margins” to “Field-Informed” Care

A lesion-centered view of DCIS ipsilateral recurrence is insufficient to explain why some patients recur despite ostensibly “clear” margins. The concept of field cancerization offers a testable alternative perspective: that recurrence risk may be influenced not only by the index DCIS lesion but also by a broader epithelial and stromal field of genetically and epigenetically altered cells, the existence of which may predate tumor formation and/or be amplified by the tumor itself. The genesis of such a field is multi-factorial (Table 1). This perspective reframes the reasons underlying recurrence as a continuum of possibilities—ranging from true residual disease at the surgical margin to “new primary” events emerging from a primed field—and is consistent with clinical observations that margin width and histology alone often fail to capture risk accurately. In other words, the margin should be looked at as a spatially graded biological compartment whose properties may provide greater prognostic insight than margin width alone, with the goal of eventual incorporation into personalized DCIS management models.

The concept of pre-tumorigenic field cancerization shines the spotlight on vulnerabilities that exist before DCIS emerges. It encompasses exposomic, hormonal, inflammatory, and tissue-context factors that may silently “prepare” ductal epithelium for neoplastic transformation long before DCIS is detectable. Environmental endocrine disruptors, diet, alcohol use, and BMI can reconfigure endocrine signaling, inflammation levels, and tumor-suppressive pathways, potentially creating a permissive milieu for emergence and expansion of clones of mutant cells. Within the breast itself, mammographic density and cyclical hormonal exposure associated with reproductive history may influence ductal epithelial and progenitor/stem cell behavior, enabling survival and dissemination of early aberrations across a broader ductal field. For recurrence prevention, these observations highlight hitherto overlooked opportunities: integrating exposure history and host factors with tissue biomarkers could help identify patients who may warrant closer surveillance, chemopreventive strategies, or intensified local therapy—while supporting de-escalation in those with less altered fields once prospectively validated.

Viral mechanisms may further intensify field vulnerability by driving chronic inflammation, stromal remodeling, and system-wide immune dysregulation. Viral-associated cytokine programs (e.g., IL-6, TNF-α) can promote ECM remodeling, fibroblast activation, angiogenesis, and immune exhaustion. Studies can be designed to evaluate whether prior viral infections serve as causal drivers, co-factors, or markers of an immunologically permissive microenvironment from which mutant cells may emerge. We assert that a near-term research priority should be to determine whether viral/immune patterns are reproducibly prognostic—and whether any risk they may confer can be modified reliably in a clinical setting. Research should also be directed toward uncovering virus-specific field effects, as a growing body of evidence suggests that even common RNA virus infections—such as influenza and SARS-CoV-2 infections—may contribute to BC progression and adverse clinical outcomes [120].

The concept of post-tumorigenic field cancerization emphasizes that the primary lesion can reshape adjacent “normal” tissue through paracrine signaling, stromal reprogramming, vascular remodeling, and exosome-mediated transfer of oncogenic cargo. Several strands of evidence support the possibility that DCIS-adjacent tissue can harbor abnormalities even when it appears histologically normal. These include microenvironmental changes such as increased vascular features, and exosome-mediated delivery of oncogenic DNA/RNA capable of inducing epigenetic reprogramming in margin cells. At the cellular level, genomic instability signatures—including AI and LOH—have been repeatedly detected in margin tissue, alongside extensive centrosome amplification, telomere shortening, and telomerase activation closer to the lesion. Collectively, these processes provide a plausible biological basis for recurrence after excision of the index tumor: the primary lesion may be removed, but a remodeled field can remain poised to re-initiate disease.

**Figure 3:**
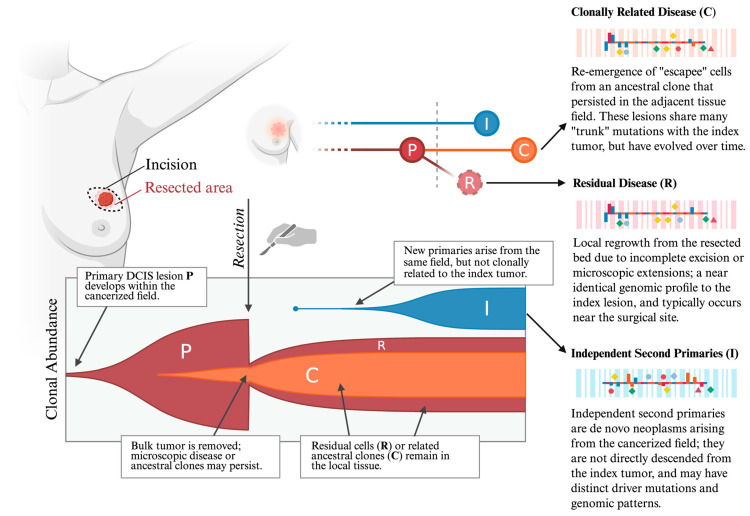
Biologically distinct mechanisms underlying ipsilateral events after DCIS excision. The figure uses fish plots to depict three evolutionary pathways to ipsilateral recurrence within a cancerized field. First, residual disease, which represents local regrowth of the index tumor due either to incomplete surgical excision or to occult microscopic extensions that escape detection during routine, purely two-dimensional histopathological evaluation. Second, clonally related recurrence, in which dispersed or field-resident descendants of the ancestral tumor clone persist within adjacent tissue, continue to evolve under selective pressures, and later re-emerge as a genetically related but molecularly diverged lesion. Third, independent second primaries, which arise de novo within a preconditioned epithelial field that harbors genomic or epigenetic vulnerability, reflecting parallel evolution rather than direct clonal continuity with the original tumor. Figure created using BioRender.

Overall, we conceptualize ipsilateral recurrence after DCIS as arising from three biologically distinct sources (Figure 3). First, from residual disease, which reflects incomplete removal of the index lesion, either because tumor cells remained in the resection bed or because conventional 2-dimensional histologic evaluation may have failed to capture all microscopic extensions within a three-dimensional margin. Ipsilateral recurrence due to residual disease is expected to recur near the surgical bed, and often within a shorter interval. Second, from clonally related recurrence, in which “escapee” (disseminated) cells of an ancestral tumor clone persist unnoticed within the adjacent tissue field and subsequently expand, giving rise to a second lesion that is genetically related to the index lineage. This second lesion may arise later and/or lead to multifocal recurrence. Third, independent second primaries, which are usually genetically distinct neoplasms that arise de novo within a preconditioned epithelial field and may share environmental or host influences with the index tumor but mostly lack direct clonal continuity. These “second primary” lesions may occur at variable distances from the original site, even when the initial lesion was completely excised. Operationally, it is possible for these categories to be distinguished using spatially resolved genomic profiling: residual disease and clonally related recurrences would be expected to share a significant number of trunk alterations with the index lesion (but differ by additional branch mutations), whereas independent second primaries would be expected to show largely distinct driver mutations and non-overlapping copy number alteration patterns compared to those in the index lesion. These distinctions are clinically meaningful as margin width is most directly relevant to residual disease, whereas clonally related recurrence and independent second primaries implicate biologically altered yet histologically normal tissue beyond the resection margin. This framework helps explain why “clear margins” do not uniformly predict outcome and why a single universal margin width may not provide equivalent risk reduction across patients.

These three biological sources of recurrence need not be viewed as discrete endpoints, but rather as outcomes emerging along a spectrum of field burden. At one end of this spectrum lies minimal or spatially confined alteration, in which recurrence risk is primarily driven by residual disease and may be effectively mitigated by adequate excision along conventional margins and appropriate adjuvant therapy. At the opposite end lies a specially extensive or genomically unstable field, in which recurrence may arise independently of margin width and may be less responsive to purely surgical solutions. Also present is an intermediate state in which clonally related recurrence reflects persistence of an ancestral lineage within a partially remodelled field. Conceptualizing recurrence along a field-burden gradient reframes margin width as one modifier of risk that may be tuned in clinical settings, rather than being viewed as a universal surrogate for biological adequacy.

Table 2a,b presents a future-oriented framework that integrates recurrence biology with plausible risk-adapted management approaches, highlighting (i) clinical management considerations that warrant prospective evaluation, and (ii) field amplifiers that modify risk across recurrence categories.

**Figure 4:**
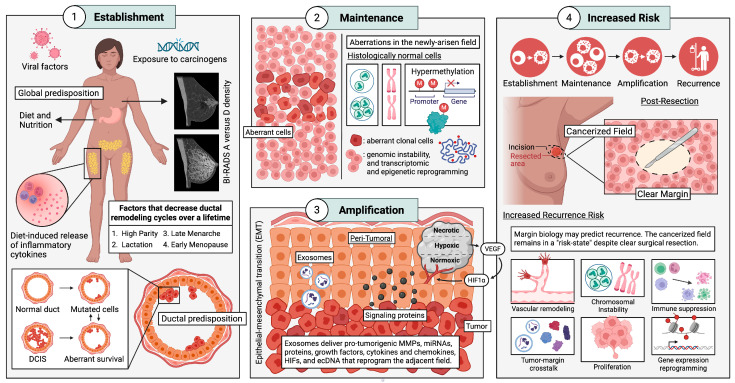
A unified model of the developmental stages of ductal field cancerization that links etiology to recurrence risk. Field cancerization is depicted as a dynamic biological state that can be consecutively established, maintained, and amplified after tumor initiation, ultimately culminating in an increase in recurrence risk. (**1**) Establishment (pre-tumorigenesis): Pre-tumorigenic influences—including endogenous factors (e.g., hormonal cycling, mammographic density), exogenous exposures (e.g., diet, endocrine disruptors), viral or immune perturbations, and inherited susceptibility—promote survival and expansion of aberrant ductal epithelial/stem cell-progenitor clones within histologically normal tissue. (**2**) Maintenance: The altered field persists as a stable niche and may expand through ongoing epithelial turnover and clonal selection, resulting in more cells exhibiting centrosome amplification, genomic instability (e.g., allelic imbalance/loss of heterozygosity), epigenetic and transcriptomic drift, and immune exhaustion in tumor-adjacent tissue, despite ostensibly benign histology. (**3**) Amplification (post-tumorigenesis): Following DCIS formation, tumor and tumor-microenvironment crosstalk (e.g., cytokines, hypoxia, stromal remodeling, extracellular vesicles/exosomes) further conditions adjacent margins by reinforcing pro-tumorigenic programs, immune modulation, ECM remodeling, and additional genetic/epigenetic perturbations, thereby expanding and intensifying changes in peri-tumoral cells. (**4**) Enhancement of recurrence risk: Recurrence risk emerges when residual malignant clones or descendants of escapee cells from the primary lesion or independent clones within the primed field cross a presumed “risk-state” threshold (e.g., heightened proliferation and CIN, vascular and immune remodeling), resulting in ipsilateral DCIS or invasive recurrence. Figure created using BioRender.

Additionally, we propose a unifying framework (Figure 4) in which field cancerization is represented as a dynamic biological state that can be (i) established, (ii) maintained, and (iii) amplified after tumorigenesis, and ultimately (iv) lead to a potentially measurable enhancement of recurrence risk. This model integrates the diverse mechanisms discussed above into a coherent sequence that explains how a field arises and could potentially persist even beyond an excised lesion. In the establishment phase, which predates the existence of any tumor, repeated physiological and exposomic pressures may create opportunities for aberrant epithelial stem/progenitor clones to survive and expand. A clear example is cyclical mammary ductal remodeling across the reproductive lifespan, which normally clears damaged cells but can inadvertently select for mutated lineages that escape clearance and undergo clonal expansion, creating a permissive epithelial landscape. The field is also spatially dynamic, and can expand under conditions of inherited predisposition, as BRCA1/2-associated genomic instability can render broader tissue compartments vulnerable to independent field “establishment.” At this stage, the field may remain undetectable by routine histology despite being biologically primed for neoplasia. Once established, the field is maintained through continued epithelial turnover coupled with persistence and selection for clones that tolerate stress, evade clearance, and progressively drift toward pro-tumorigenic states. The maintenance stage conceivably involves microenvironmental accommodation—immune tolerance/exhaustion and stromal permissiveness—such that the field becomes a stable niche rather than a transient perturbation. In the post-tumorigenic amplification stage, tumor-derived extracellular vesicles and soluble factors potentially deliver oncogenic cargo (miRNAs, pro-survival proteins), promote ECM remodeling, immune modulation, EMT, and induce genomic instability phenotypes in adjacent histologically normal cells. These post-tumor influences provide a biological basis for recurrence even when the visible lesion is removed, because the adjacent tissue has been functionally remodeled into a pro-tumorigenic field. Therefore, it is plausible that recurrence risk increases when field alterations cross a presumed threshold into an “enhanced risk-state,” potentially evaluable by biomarkers—e.g., centrosome amplification, genomic instability, telomere attrition, epigenetic remodeling, and immune suppression—rather than by margin status alone.

From a clinical perspective, this implies that further research into recurrence risk should be assessed using two orthogonal dimensions: (i) lesion-specific features and (ii) field-specific metrics. Lesion-specific features capture intrinsic tumor biology, while field-specific metrics quantify the spatial extent and severity of biologically altered, yet histologically normal, tissue. Integrating these dimensions could allow the development of stratification models relating to biologically defined risk groups—for example, patients with low lesion aggressiveness and low field burden who may be candidates for de-escalation, versus patients with limited lesion size but possess highly distributed fields with a high degree of instability who may derive benefit from intensified local or systemic therapy despite otherwise “clear” margins. Table 3 operationalizes this framework, offering potential quantifiable biomarkers for each developmental stage and proposing the incorporation of these biomarkers into individualized risk management models.

A field-informed model, therefore, suggests that different recurrence mechanisms may warrant different management strategies. Residual disease may be most effectively addressed through surgical resection and radiotherapy. Clonally related recurrence may require strategies that target persistent lineage-specific vulnerabilities, including endocrine therapy or targeted systemic approaches. Independent second primaries may be more sensitive to interventions that modify the broader tissue environment, such as risk-reducing endocrine therapy, anti-inflammatory approaches, or intensified surveillance. Without distinguishing among these mechanisms, management decisions risk conflating biologically distinct processes under a single margin-based metric.

Today, DCIS management decisions—extent of surgery, need for RT, endocrine therapy, or surveillance intensity—are often made with incomplete information about tumor-adjacent tissue biology. Incorporating prognostically informative field-derived biomarkers into recurrence risk models could improve risk stratification and help resolve persistent dilemmas such as the issue of margin adequacy. A field-informed approach could reconceptualize tumor margin assessment as a spatial biology analysis conducted along a gradient (e.g., 2 mm to 2 cm) with the goal of quantifying the prevalence and severity of centrosome amplification, identifying prognostic gene expression signatures, measuring genomic instability, immune suppression, telomere attrition, and epigenetic alterations. Defining quantitative thresholds for these parameters–individually or as composite indices–would permit classification of patients into biologically grounded risk tiers that could inform margin adequacy, need for adjuvant therapy, or eligibility for surveillance-based approaches. Such an in-depth and granular assessment would represent a significant shift from the “margin is clear” versus “margin is positive” binary, but it will require standardized sampling and prospective validation. Concordantly, adjuvant therapy intensity would need to be matched to models that incorporate prognostic features of the DCIS lesion as well as field-informed recurrence risk prognosticators, if field measures are shown to add reproducible prognostic information in prospective cohorts. Ultimately, patients with high-risk tumors and fields may benefit from intensified local control (e.g., adjuvant RT) and/or systemic strategies, while those with low-risk tumors and fields could be candidates for de-escalation. Longitudinal studies that track how the tumor-adjacent field develops over time could clarify the long-term role of field alterations and their impact on risk stratification. From a clinical viewpoint, we could also benefit from studies that examine tumorigenic associations with exposure to endocrine disruptors and xenoestrogens. These potential findings could have a significant impact on public education and inform hormonal contraception and fertility counseling.

We acknowledge that much of the current evidence linking field cancerization to DCIS recurrence remains associative, drawn largely from retrospective cohorts and small series. DCIS itself is biologically diverse with respect to grade, architecture, extent, and molecular subtype; consequently, “field” features described across studies may not represent a single, uniform biology but rather multiple field states that differ by patient and lesion context. In addition, sampling bias is a pervasive constraint: margin specimens are often taken opportunistically, vary in distance from the index lesion, and can be contaminated by normal adipose-rich tissue or by under-sampled epithelial compartments—limitations that can dilute or distort detection of ductal field alterations. Many molecular assays used to characterize margins also rely on bulk measurements with limited cellular resolution, making it difficult to disentangle whether observed signals arise from altered ductal epithelium, reactive stroma, or admixture effects. Another central interpretive challenge is distinguishing true field effects from occult residual disease or microscopic multifocality. Table 4 highlights (a) methodological limitations that must be addressed before field measures can be used for patient-level decision-making, and (b) potential mitigation strategies that can be incorporated into study designs.

Another critical research implication of the concept of field cancerization is that tumor-adjacent or ipsilateral margin tissue is often a suboptimal “healthy control tissue,” given the extensively catalogued alterations and abnormalities present in margin cells, and documented in the literature. Using tumor-adjacent tissue as a baseline for comparisons can obscure true differences, distort biomarker discovery, and produce misleading conclusions. Future studies should preferentially use contralateral breast tissue (when ethically and practically feasible), reduction mammoplasty specimens, or carefully characterized “distance-mapped” samples to establish valid baselines. At minimum, ipsilateral margin tissue should be explicitly labeled as “field tissue” rather than “normal,” to avoid over-interpreting it as representative of healthy tissue.

To truly change practice, there is a strong need to assemble integrated, high-resolution datasets that capture the details of the “phenome” and “genome” of the field, the patient’s history of exposures to endocrine disruptors, their diet, BMI, reproductive factors, and past viral infections, alongside clinicopathologic and mammographic density data, and look for associations between the afore-mentioned factors and clinical outcomes. Towards this end, a standardized collection of tissues at defined distances from DCIS (e.g., 2 mm, 5 mm, 1 cm, 2 cm), standardized fixation and processing, and annotation, would be imperative. Multi-omic margin profiling approaches could include single-cell and spatial transcriptomics, methylation/ATAC-seq, and targeted genomic instability measures (AI/LOH), paired with immune phenotyping. Inclusion of metrics of vascular remodeling, fibroblast activation programs, and local microbiome signatures in relation to field biomarkers and recurrence could improve the evaluation of recurrence-associated field features and help test whether multi-compartment field measures improve prediction. We anticipate that the identification and validation of robust and measurable biomarkers that are prognostically informative in tumor-adjacent tissues, in relation to meaningful endpoints, could support improved patient care, fewer unnecessary escalations for low-risk disease, earlier identification of patients at high risk of recurrence, and new preventive/adjuvant strategies aimed at the biology of the field that remains after the primary lesion is removed.

Evidence indicates that ipsilateral recurrence after DCIS is not fully explained by margin width or histologic features alone, that tumor-adjacent tissue can harbor reproducible genomic and microenvironmental alterations despite appearing histologically normal, and that spatial genomic profiling can distinguish residual disease from clonally related recurrences and independent second primaries. Plausible but not yet definitively proven mechanisms include the establishment of a pre-tumorigenic epithelial field shaped by exposomic, hormonal, inflammatory, and possibly viral influences, as well as post-tumorigenic amplification of field alterations through paracrine signaling, extracellular vesicles, stromal reprogramming, and immune modulation. At present, clinically actionable implications are limited to continued emphasis on adequate surgical excision for residual disease risk, careful interpretation of margin tissue as biologically altered rather than truly normal, and integration of established clinicopathologic and host factors into individualized recurrence discussions, while field-derived biomarkers remain investigational. Future research must determine whether standardized spatial sampling and multi-omic profiling of tumor-adjacent tissue yield reproducible, prospectively validated biomarkers that add prognostic value beyond current models and can guide escalation or de-escalation of local and systemic therapy.

## Figures and Tables

**Table 1:** Multifactorial drivers of field cancerization before and after tumor initiation.

Potential Drivers of Recurrence (Examples)	Mechanistic Link to Field Cancerization	Field Alterations/Measurable Biomarkers (Examples)	How It Could Inform Clinical Decisions (Examples)
Endocrine disruptors (phthalates, bisphenols)	Estrogen-pathway perturbation promotes proliferative signaling and inflammatory conditioning	Estrogen receptor and progesterone receptor signaling/activation signatures; inflammatory cytokine programs; epigenetic shifts; proliferation markers	Identify hormone-driven field activity → consider endocrine prevention/optimization; tighter surveillance; emphasize margin profiling even if histology is negative
Alcohol/acetaldehyde exposure	DNA damage. Oxidative stress may subsequently expand into a field with damaged DNA (especially with chronic exposure)	DNA damage response (DDR) signatures; oxidative stress markers; genomic instability signatures	High DDR/instability in margins → may support escalated local control (radiotherapy benefit) and closer follow-up
Dietary protective factors (soy/isoflavones)	Differentiation-promoting and potentially anti-inflammatory effects may limit field expansion	Differentiation-associated gene programs; reduced proliferative/inflammatory signatures	Low-risk field biology → may support de-escalation strategies when lesion pathology is borderline
High glycemic index/hyperinsulinemia	Growth-factor–like signaling fosters tumor-favorable microenvironment and may strengthen or expand a cancerized field	Insulin/IGF signaling signatures; proliferation programs; stromal activation	Pro-growth field biology → may support more aggressive local control (radiotherapy) and monitoring for recurrence
Inflammatory cytokine milieu (obesity, distress, chronic inflammation)	Cytokine-rich environment supports survival and immune evasion	IL-6/IL-8/TGF-β programs; immune suppression/exhaustion signatures; CAF activation; ECM remodeling	Inflamed + immunosuppressed margins → consider radiotherapy benefit; avoid de-escalation; increase surveillance intensity
High mammographic density	Collagen deposition/stiffness + stromal architecture creates a permissive, cancerization-prone microenvironment	Collagen alignment, stiffness-associated programs; CAF signatures; increased risk	Dense breasts + high-risk field signatures → could potentially justify supplemental imaging, more intensive surveillance, potentially lower threshold for RT
Ductal remodeling (menarche/menopause, parity, involution)	Cyclical turnover may inadvertently expand surviving mutant clones	Clonal expansion markers; mutation burden/AI/LOH in normal-appearing epithelium; proliferative programs	Younger/premenopausal + high-risk field biomarkers → avoid de-escalation; tailor surveillance and adjuvant decisions
Viral persistence (EBV, HHV-5/HCMV)—speculative for DCIS recurrence	Immune dysregulation + chronic inflammation + pathway perturbation may amplify field survival and remodeling	Immune exhaustion signatures; inflammatory cytokines; TGF-β/MMP activation; viral burden/latency markers	If validated: viral/immune-high-risk field state could justify closer surveillance and inform immunologic risk models
Post-tumorigenesis alterations in adjacent tissue	Peritumoral histological normal tissue has been shown to acquire genetic/epigenetic/telomeric abnormalities in a distance-dependent gradient; microenvironment within the margin gains increased vascular density, and immune-rich stroma	AI/LOH; CNV burden; ERBB2 copy gain; methylation changes; transcriptomic shift; telomere shortening/telomerase expression; Micro vessel/periductal vascular density; immune infiltration patterns	Potentially high-risk margin biology despite clear histology → may support wider re-excision, RT benefit, and higher-intensity surveillance
Centrosome amplification (CA/CAS)	Early driver of chromosomal instability; present in tumor-adjacent histological normal cells; prognostic for recurrence	Numerical/structural CA; CIN programs; recurrence-associated CAS	High CA/CIN field state → higher recurrence risk → may support RT, careful margin management; avoid de-escalation
Exosomes/miRNAs/cargo (HIFs, MMPs, cytokines, survivin, ecDNA)	Tumor-to-field communication reprograms adjacent tissue; supports EMT, immune suppression, angiogenesis, survival	Exosomal miRNA signatures; cytokine/MMP programs; survivin presence; ecDNA-related oncogene dosage effects	If validated: non-invasive field activity tracking → informs surveillance intensity and recurrence-risk updates over time

**Table 2:** (**a**) Integrated model linking recurrence biology to risk-adapted management approaches that merit further study. (**b**) Cross-Cutting Field Amplifiers Modifying Recurrence Probability Across Categories.

**(a)**						
**Recurrence Category**	**Field Biology Driver**	**Expected Molecular Pattern in Margin/Field**	**Clonality Signature**	**Time Horizon**	**Projected Risk Stratification**	**Investigational Implications**
Residual disease	Incomplete excision of clonally identical tumor cells	Identical driver mutations; concordant AI/LOH; ERBB2 copy concordance; high centrosome amplification localized within 0–2 mm margin	Genomically identical to index lesion	Early (short interval)	High	Consider re-excision; strong radiotherapy recommendation; intensified early surveillance
Clonally related recurrence	Pre-existing cancerized ductal field with evolutionary divergence	Shared ancestral mutations with subclonal divergence; distance-dependent AI gradient; telomere shortening; CA in histologically normal epithelium; epigenetic field signatures	Partially shared ancestry with genomic divergence	Intermediate	Intermediate–High	Margin molecular profiling; radiotherapy consideration; avoid de-escalation in high-field-burden cases
Independent second primary tumor	Diffuse genomic instability or systemic predisposition (e.g., BRCA/HRD background)	Distinct mutation profile from index lesion; global AI/CNV burden; HRD signature; widespread epigenetic dysregulation	Genomically distinct from primary tumor	Late/Long-term	Variable but sustained recurrence risk	Genetic counseling/testing; systemic risk-reduction strategies; bilateral imaging surveillance; preventive interventions
(**b**)						
**Field Amplifier**		**Mechanistic Role**	**Effect on Recurrence Categories**			**Translational Considerations**
Inflammatory/cytokine-enriched stroma		Has been demonstrated to promote survival, immune evasion, and clonal expansion within field	Increases likelihood of residual disease persistence and clonal expansion			Evaluate radiotherapy; increase surveillance intensity
Centrosome amplification		Established driver of chromosomal instability and subclonal diversification	Promotes progression of residual disease, and can additionally drive clonally related and independent events			Test integration of CA/CIN metrics into recurrence nomograms; avoid de-escalation if elevated
Exosome-mediated signaling		Potential horizontal transfer of oncogenic miRNAs, proteins, survivin, ecDNA	Expands field permissiveness across all categories			Future liquid biopsy monitoring; closer follow-up in high exosomal burden states
High mammographic density/stromal stiffness		Collagen deposition and mechanical signaling have been shown to promote field permissiveness	Amplifies local recurrence risk independent of margin width			Assess need for supplemental imaging; risk-adapted radiotherapy consideration

**Table 3:** Translating stages of field cancerization into measurable biomarkers and possible clinical decision points.

Field Cancerization Stage	Core Biological Processes (Integrated Model)	Example Assays/Readouts	How It Translates to Recurrence Risk and Aids in Risk Management
(1) Establishment [pre-tumorigenesis]	Selection and expansion of aberrant epithelial stem/progenitor clones during cyclic remodeling and cumulative exposures. Predisposition widens susceptible tissue compartment.	Targeted panels for clonal mutations and CNAs. Methylation panels in benign epithelium. Spatial sampling across ducts.	May help identify individuals with a biologically expanded at-risk field. Such information could support closer surveillance or risk counseling, although prospective validation is required.
(2) Maintenance [persistence and spatial spread]	Ongoing genomic instability, and transcriptional and epigenetic reprogramming in histologically normal peri-tumoral cells, enabling spatial persistence of altered clones.	CA/AI/LOH assays Methylation and ATAC panels Spatial transcriptomics EMT/ECM expression signatures	May highlight margins with biologic alterations, supporting further investigation of risk beyond margin width alone in selected contexts.
(3) Amplification [post-tumorigenic tumor-margin crosstalk]	Tumor-derived exosomes, cytokines, and hypoxia signaling remodel adjacent tissue through delivery of miRNAs, ecDNA, and proteins. Immune and stromal reprogramming. EMT/stemness.	EV/exosome miRNA panels. Cytokine and chemokine profiles. Stromal signatures. Immune phenotyping and CA quantification (IHC/spatial).	Provides a conceptual framework for considering tumor-field interactions in adjuvant strategy development, although clinical utility remains to be prospectively validated.
(4) Increased Recurrence Risk [clinical conversion: risk state → recurrence event]	Quantitative thresholds of instability and a permissive microenvironment yield recurrence (residual, clonally related, or new primary along a continuum).	Periductal vascular density quantification. Centrosome assays. Telomere metrics. Integrated multi-omic margin profiling.	May contribute to the development and refinement of multi-factorial risk models incorporating margin-derived biomarkers. If validated, such models could inform risk-adapted decisions regarding radiotherapy, re-excision, or surveillance intensity while identifying patients appropriate for de-escalation.

**Table 4:** Current gaps and limitations in field-recurrence studies and potential mitigation strategies.

Limitation	Why It Matters	Practical Mitigation (Study Design/Assay)
Biological heterogeneity of DCIS Some examples: Heterogeneity in grade, architecture, extent (radial span, number of ducts involved, unifocal vs. multifocal vs. multicentric, and segmental vs. diffuse growth), molecular subtype	Tumor-derived “field” signals may be diverse, and pooling across heterogeneous DCIS samples can blur associations or confound biomarker discovery	Stratify analyses by DCIS grade/subtype/extent Adjust for key covariates Validate within each stratum and across independent cohorts
Sampling bias (where sample collected may not represent the true biology of the tumor or the margin area), inconsistencies in samples collected, and limited spatial resolution of molecular assays. Some examples: Opportunistic sampling (using whatever tissue is available after diagnostic processing rather than using only what is prospectively defined) Samples derived from variable distances from margin Variable levels of epithelial content dilution by adipose: breast margins often contain significant amounts of fat, because of which ductal epithelium may represent a small fraction of the sample	Field effects are likely distance dependent. Without consistent mapping of sample’s distance from tumor margin, comparisons across patients or studies become unreliable Bulk molecular assays may detect signals dominated by adipocytes and stroma Non-standardized sampling can produce apparent “field differences” driven by tissue composition (stromal/purity effects) rather than by true differences in biology; cell-type admixture can masquerade as field biology	Standardize distance-mapped sampling (e.g., tumor bed, 0–2 mm, 2–10 mm, 1–2 cm) and sample processing steps Record exact distances and specimen orientation Use laser-capture microdissection of ducts/epithelium to isolate pure fractions from complex tissues Perform single-cell or spatial transcriptomics Perform deconvolution with purity estimates, and report cell-type proportions
Difficulty distinguishing field effects from occult residual disease or microscopic multifocality	Shared genomic alterations between index tumor and recurrence could reflect occult residual disease rather than a preconditioned field; misclassification weakens mechanistic inference. Residual disease tends to recur at or very near the surgical bed, and clonally related but field-resident clones may recur slightly offset from the bed but within the same quadrant. By contrast, new independent primaries may arise more distally within the ipsilateral breast. So, it is important to map precisely where the recurrence arises relative to the original tumor bed, the original margin orientation, and distance from the surgical cavity.	Combine rigorous pathology with multi-region sampling Map clones phylogenetically (by identifying trunk vs. branch alterations) Integrate information about the location of the surgical bed as well as time-to-event (recurrence) data; model recurrence as a time-dependent process. Stratify analyses by early vs. late recurrence Use prospective designs for studies. Prospective studies allow you to determine whether field biomarkers predict recurrence independent of margin status, whether recurrence arises at pre-characterized “high-risk field zones”, whether certain field signatures predict late but not early recurrence Employ prospective, distance-mapped sampling with predefined recurrence classification criteria to reduce misattribution of occult residual disease to field effects
Retrospective study designs and use of small cohorts Multiple testing or overfitting of candidate biomarkers	Inflates effect sizes and reduces reproducibility Optimized cut-points may not generalize	Enroll patients prospectively at time of surgery Predefine margin sampling strategy (distance-mapped) Pre-specified endpoints (ipsilateral DCIS vs. IDC; early vs. late recurrence) Follow patients longitudinally Pre-registered analysis plans to minimize post hoc selection bias K-fold cross-validation and external validation to confirm robustness and generalizability of model (i.e., test the biomarker/model in an independent cohort preferably from another institution, with similar but not identical patient characteristics) Use penalized regression techniques in high-dimensional settings
Confounding effects of treatment and clinical management (radiation therapy/endocrine therapy; re-excision)	Treatment effects can confound associations between field markers and recurrence, limiting causal interpretation	Model treatments as covariates and test interactions
Technical variation in fixation/processing/batch effects	Batch effects can dominate subtle margin signals Batch effects can undermine cross-cohort comparisons	Standardize fixation/processing Include technical replicates Randomize batches Apply batch correction Report sample exclusion criteria

## Data Availability

The original contributions presented in this study are included in the article. Further inquiries can be directed to the corresponding author.

## References

[1] Kalwaniya D.S., Gairola M., Gupta S., Pawan G., Gurivelli P. (2023). Ductal Carcinoma in Situ: A Detailed Review of Current Practices. Cureus.

[2] Chang Y.-W., Ryu J.K., An J.K., Choi N., Park Y.M., Ko K.H., Han K. (2025). Artificial intelligence for breast cancer screening in mammography (AI-STREAM): Preliminary analysis of a prospective multicenter cohort study. Nat. Commun..

[3] Siegel R.L., Miller K.D., Fuchs H.E., Jemal A. (2022). Cancer statistics. CA Cancer J. Clin..

[4] Siegel R.L., Giaquinto A.N., Jemal A. (2024). Cancer statistics, 2024. CA A Cancer J. Clin..

[5] Co M., Cheng K., Yeung Y., Lau K., Qian Z., Wong C., Wong B., Sin E., Wong H., Ma C. (2023). Clinical Outcomes of Conservative Treatment for Low-Risk Ductal Carcinoma in Situ: A Systematic Review and Pooled Analysis. Clin. Oncol..

[6] Williams L.J., Kunkler I.H., Taylor K.J., Dunlop J., Piper T., Caldwell J., Jack W., Loane J.F., Elder K., Bartlett J.M.S. (2024). Postoperative radiotherapy in women with early operable breast cancer (Scottish Breast Conservation Trial): 30-year update of a randomised, controlled, phase 3 trial. Lancet Oncol..

[7] Withrow D.R., Morton L.M., Curtis R.E., Schonfeld S.J., de González A.B. (2017). Radiotherapy for ductal carcinoma in situ and risk of second non-breast cancers. Breast Cancer Res. Treat..

[8] Wadsten C., Wennstig A.-K., Garmo H., Nilsson G., Blomqvist C., Holmberg L., Fredriksson I., Wärnberg F., Sund M. (2018). Risk of ischemic heart disease after radiotherapy for ductal carcinoma in situ. Breast Cancer Res. Treat..

[9] Solin L.J., Gray R., Hughes L.L., Wood W.C., Lowen M.A., Badve S.S., Baehner F.L., Ingle J.N., Perez E.A., Recht A. (2015). Surgical Excision Without Radiation for Ductal Carcinoma in Situ of the Breast: 12-Year Results From the ECOG-ACRIN E5194 Study. J. Clin. Oncol..

[10] Rudloff U., Jacks L.M., Goldberg J.I., Wynveen C.A., Brogi E., Patil S., Van Zee K.J. (2010). Nomogram for Predicting the Risk of Local Recurrence After Breast-Conserving Surgery for Ductal Carcinoma In Situ. J. Clin. Oncol..

[11] Milonas D., Venclovas Z., Muilwijk T., Jievaltas M., Joniau S. (2020). External validation of Memorial Sloan Kettering Cancer Center nomogram and prediction of optimal candidate for lymph node dissection in clinically localized prostate cancer. Central Eur. J. Urol..

[12] Choi J.D.W., Hughes T.M.D., Marx G., Boyages J., Rutovitz J., Hasovits C., Parasyn A., Edirimanne S., Ngui N.K. (2022). The Utility of the Oncotype DX Test for Breast Cancer Patients in an Australian Multidisciplinary Setting. Breast J..

[13] Shah C., Bremer T., Cox C., Whitworth P., Patel R., Patel A., Brown E., Gold L., Rock D., Riley L. (2021). The Clinical Utility of DCISionRT^®^ on Radiation Therapy Decision Making in Patients with Ductal Carcinoma In Situ Following Breast-Conserving Surgery. Ann. Surg. Oncol..

[14] Pawloski K.R., Tadros A.B., Sevilimedu V., Newman A., Gentile L., Zabor E.C., Morrow M., Van Zee K.J., Kirstein L.J. (2021). Patterns of invasive recurrence among patients originally treated for ductal carcinoma in situ by breast-conserving surgery versus mastectomy. Breast Cancer Res. Treat..

[15] Tamburelli F., Maggiorotto F., Marchiò C., Balmativola D., Magistris A., Kubatzki F., Sgandurra P., Di Virgilio M.R., Regge D., Montemurro F. (2020). Reoperation rate after breast conserving surgery as quality indicator in breast cancer treatment: A reappraisal. Breast.

[16] Co M., Fung M.W.Y., Kwong A. (2024). Surgical margin and local recurrence of ductal carcinoma in situ. Cancer Treat. Res. Commun..

[17] Schmitz R.S.J.M., Belt-Dusebout A.W.v.D., Clements K., Ren Y., Cresta C., Timbres J., Liu Y.-H., Byng D., Lynch T., A Menegaz B. (2023). Association of DCIS size and margin status with risk of developing breast cancer post-treatment: Multinational, pooled cohort study. BMJ.

[18] Chien J.-C., Huang W.-T., Shih L.-C., Liu W.-C., Chen Y.-C., Chou K.-J., Shiue Y.-L., Lin P.-C. (2022). Local treatment options for young women with ductal carcinoma in situ: A systematic review and meta-analysis comparing breast conserving surgery with or without adjuvant radiotherapy, and mastectomy. Breast.

[19] Gadaleta E., Thorn G.J., Ross-Adams H., Jones L.J., Chelala C. (2022). Field cancerization in breast cancer. J. Pathol..

[20] Wilson G.M., Dinh P., Pathmanathan N., Graham J.D. (2022). Ductal Carcinoma in Situ: Molecular Changes Accompanying Disease Progression. J. Mammary Gland. Biol. Neoplasia.

[21] Moumen M., Chiche A., Cagnet S., Petit V., Raymond K., Faraldo M.M., Deugnier M.-A., Glukhova M.A. (2011). The mammary myoepithelial cell. Int. J. Dev. Biol..

[22] Siegel R.L., Miller K.D., Jemal A. (2020). Cancer statistics, 2020. CA Cancer J. Clin..

[23] Farabegoli F., Champeme M., Bieche I., Santini D., Ceccarelli C., Derenzini M., Lidereau R. (2002). Genetic pathways in the evolution of breast ductal carcinoma in situ. J. Pathol..

[24] Bièche I., Lidereau R. (1995). Genetic alterations in breast cancer. Genes, Chromosom. Cancer.

[25] Russell T.D., Jindal S., Agunbiade S., Gao D., Troxell M., Borges V.F., Schedin P. (2015). Myoepithelial Cell Differentiation Markers in Ductal Carcinoma in Situ Progression. Am. J. Pathol..

[26] Mardekian S.K., Bombonati A., Palazzo J.P. (2016). Ductal carcinoma in situ of the breast: The importance of morphologic and molecular interactions. Hum. Pathol..

[27] Rohilla M., Bal A., Singh G., Joshi K. (2015). Phenotypic and Functional Characterization of Ductal Carcinoma In Situ–Associated Myoepithelial Cells. Clin. Breast Cancer.

[28] Allen M.D., Thomas G.J., Clark S., Dawoud M.M., Vallath S., Payne S.J., Gomm J.J., Dreger S.A., Dickinson S., Edwards D.R. (2014). Altered Microenvironment Promotes Progression of Preinvasive Breast Cancer: Myoepithelial Expression of αvβ6 Integrin in DCIS Identifies High-risk Patients and Predicts Recurrence. Clin. Cancer Res..

[29] Toss M., Miligy I., Gorringe K., Mittal K., Aneja R., Ellis I., Green A., Rakha E. (2019). Prognostic significance of cathepsin V (CTSV/CTSL2) in breast ductal carcinoma in situ. J. Clin. Pathol..

[30] Toss M.S., Miligy I.M., Gorringe K.L., AlKawaz A., Khout H., Ellis I.O., Green A.R., Rakha E.A. (2018). Prolyl-4-hydroxylase A subunit 2 (P4HA2) expression is a predictor of poor outcome in breast ductal carcinoma in situ (DCIS). Br. J. Cancer.

[31] van Seijen M., Lips E.H., Thompson A.M., Nik-Zainal S., Futreal A., Hwang E.S., Verschuur E., Lane J., Jonkers J., Rea D.W. (2019). Ductal carcinoma in situ: To treat or not to treat, that is the question. Br. J. Cancer.

[32] Yates L.R., Gerstung M., Knappskog S., Desmedt C., Gundem G., Van Loo P., Aas T., Alexandrov L.B., Larsimont D., Davies H. (2015). Subclonal diversification of primary breast cancer revealed by multiregion sequencing. Nat. Med..

[33] Casasent A.K., Edgerton M., Navin N.E. (2016). Genome evolution in ductal carcinoma *in situ*: Invasion of the clones. J. Pathol..

[34] Wang J., Li B., Luo M., Huang J., Zhang K., Zheng S., Zhang S., Zhou J. (2024). Progression from ductal carcinoma in situ to invasive breast cancer: Molecular features and clinical significance. Signal Transduct. Target. Ther..

[35] Bray F., McCarron P., Parkin D.M. (2004). The changing global patterns of female breast cancer incidence and mortality. Breast Cancer Res..

[36] Diamanti-Kandarakis E., Bourguignon J.-P., Giudice L.C., Hauser R., Prins G.S., Soto A.M., Zoeller R.T., Gore A.C. (2009). Endocrine-Disrupting Chemicals: An Endocrine Society Scientific Statement. Endocr. Rev..

[37] Segovia-Mendoza M., Palacios-Arreola M.I., Monroy-Escamilla L.M., Soto-Piña A.E., Nava-Castro K.E., Becerril-Alarcón Y., Camacho-Beiza R., Aguirre-Quezada D.E., Cardoso-Peña E., Amador-Muñoz O. (2022). Association of Serum Levels of Plasticizers Compounds, Phthalates and Bisphenols, in Patients and Survivors of Breast Cancer: A Real Connection?. Int. J. Environ. Res. Public Heal..

[38] Ahern T.P., Broe A., Lash T.L., Cronin-Fenton D.P., Ulrichsen S.P., Christiansen P.M., Cole B.F., Tamimi R.M., Sørensen H.T., Damkier P. (2019). Phthalate Exposure and Breast Cancer Incidence: A Danish Nationwide Cohort Study. J. Clin. Oncol..

[39] Yang P.-J., Hou M.-F., Ou-Yang F., Hsieh T.-H., Lee Y.-J., Tsai E.-M., Wang T.-N. (2022). Association between recurrent breast cancer and phthalate exposure modified by hormone receptors and body mass index. Sci. Rep..

[40] Stillwater B.J., Bull A.C., Romagnolo D.F., Neumayer L.A., Donovan M.G., Selmin O.I. (2020). Bisphenols and Risk of Breast Cancer: A Narrative Review of the Impact of Diet and Bioactive Food Components. Front. Nutr..

[41] Scoccianti C., Lauby-Secretan B., Bello P.-Y., Chajes V., Romieu I. (2014). Female Breast Cancer and Alcohol Consumption. Am. J. Prev. Med..

[42] Simapivapan P., Boltong A., Hodge A. (2016). To what extent is alcohol consumption associated with breast cancer recurrence and second primary breast cancer?: A systematic review. Cancer Treat. Rev..

[43] Allen N.E., Beral V., Casabonne D., Kan S.W., Reeves G.K., Brown A., Green J., on behalf of the Million Women Study Collaborators (2009). Moderate Alcohol Intake and Cancer Incidence in Women. JNCI J. Natl. Cancer Inst..

[44] Kwan M.L., Kushi L.H., Weltzien E., Tam E.K., Castillo A., Sweeney C., Caan B.J. (2010). Alcohol Consumption and Breast Cancer Recurrence and Survival Among Women with Early-Stage Breast Cancer: The Life After Cancer Epidemiology Study. J. Clin. Oncol..

[45] Kabat G.C., Kim M., Shikany J.M., Rodgers A.K., Wactawski-Wende J., Lane D., Powell L., Stefanick M.L., Freiberg M.S., Kazlauskaite R. (2010). Alcohol Consumption and Risk of Ductal Carcinoma *In situ* of the Breast in a Cohort of Postmenopausal Women. Cancer Epidemiol. Biomark. Prev..

[46] Messina M., Hilakivi-Clarke L. (2009). Early Intake Appears to Be the Key to the Proposed Protective Effects of Soy Intake Against Breast Cancer. Nutr. Cancer.

[47] Shu X.O., Zheng Y., Cai H., Gu K., Chen Z., Zheng W., Lu W. (2009). Soy Food Intake and Breast Cancer Survival. JAMA.

[48] Nechuta S.J., Caan B.J., Chen W.Y., Lu W., Chen Z., Kwan M.L., Flatt S.W., Zheng Y., Zheng W., Pierce J.P. (2012). Soy food intake after diagnosis of breast cancer and survival: An in-depth analysis of combined evidence from cohort studies of US and Chinese women. Am. J. Clin. Nutr..

[49] Ismail A., El Awady R., Abdelsalam G., Hussein M., Ramadan S. (2018). Prognostic Significance of Serum Vitamin D Levels in Egyptian Females with Breast Cancer. Asian Pac. J. Cancer Prev..

[50] Goodwin P.J., Ennis M., Pritchard K.I., Koo J., Hood N. (2009). Prognostic Effects of 25-Hydroxyvitamin D Levels in Early Breast Cancer. J. Clin. Oncol..

[51] de Sousa Almeida-Filho B., De Luca Vespoli H., Pessoa E.C., Machado M., Nahas-Neto J., Nahas E.A.P. (2017). Vitamin D deficiency is associated with poor breast cancer prognostic features in postmenopausal women. J. Steroid Biochem. Mol. Biol..

[52] Jacobs E.T., A Thomson C., Flatt S.W., Al-Delaimy W.K., A Hibler E., A Jones L., LeRoy E.C., A Newman V., A Parker B., Rock C.L. (2011). Vitamin D and breast cancer recurrence in the Women’s Healthy Eating and Living (WHEL) Study. Am. J. Clin. Nutr..

[53] Augustin L.S., Libra M., Crispo A., Grimaldi M., De Laurentiis M., Rinaldo M., D’aiuto M., Catalano F., Banna G., Ferrau’ F. (2017). Low glycemic index diet, exercise and vitamin D to reduce breast cancer recurrence (DEDiCa): Design of a clinical trial. BMC Cancer.

[54] Redwan E.M., Linjawi M.H., Uversky V.N. (2016). Looking at the carcinogenicity of human insulin analogues via the intrinsic disorder prism. Sci. Rep..

[55] A Habel L., Daling J.R., A Newcomb P., Self S.G., Porter P.L., Stanford J.L., Seidel K., Weiss N.S. (1998). Risk of recurrence after ductal carcinoma in situ of the breast. Cancer Epidemiol. Biomark. Prev..

[56] Lee H.-M., Lee H.-J., Chang J.-E. (2022). Inflammatory Cytokine: An Attractive Target for Cancer Treatment. Biomedicines.

[57] Takeuchi Y., Gotoh N. (2023). Inflammatory cytokine-enriched microenvironment plays key roles in the development of breast cancers. Cancer Sci..

[58] Sparano J.A., O’nEill A., Graham N., Northfelt D.W., Dang C.T., Wolff A.C., Sledge G.W., Miller K.D. (2022). Inflammatory cytokines and distant recurrence in HER2-negative early breast cancer. npj Breast Cancer.

[59] Cuzick J., Otto F., Baron J.A., Brown P.H., Burn J., Greenwald P., Jankowski J., La Vecchia C., Meyskens F., Senn H.J. (2009). Aspirin and non-steroidal anti-inflammatory drugs for cancer prevention: An international consensus statement. Lancet Oncol..

[60] de Pedro M., Baeza S., Escudero M.-T., Dierssen-Sotos T., Gómez-Acebo I., Pollán M., Llorca J. (2015). Effect of COX-2 inhibitors and other non-steroidal inflammatory drugs on breast cancer risk: A meta-analysis. Breast Cancer Res. Treat..

[61] Groenvold M., Petersen M.A., Idler E., Bjorner J.B., Fayers P.M., Mouridsen H.T. (2007). Psychological distress and fatigue predicted recurrence and survival in primary breast cancer patients. Breast Cancer Res. Treat..

[62] Yan J., Chen Y., Luo M., Hu X., Li H., Liu Q., Zou Z. (2023). Chronic stress in solid tumor development: From mechanisms to interventions. J. Biomed. Sci..

[63] Martin L.J., Boyd N.F. (2008). Mammographic density. Potential mechanisms of breast cancer risk associated with mammographic density: Hypotheses based on epidemiological evidence. Breast Cancer Res..

[64] Li T., Sun L., Miller N., Nicklee T., Woo J., Hulse-Smith L., Tsao M.-S., Khokha R., Martin L., Boyd N. (2005). The Association of Measured Breast Tissue Characteristics with Mammographic Density and Other Risk Factors for Breast Cancer. Cancer Epidemiol. Biomark. Prev..

[65] McConnell J.C., O’cOnnell O.V., Brennan K., Weiping L., Howe M., Joseph L., Knight D., O’cUalain R., Lim Y., Leek A. (2016). Increased peri-ductal collagen micro-organization may contribute to raised mammographic density. Breast Cancer Res..

[66] Vinnicombe S. (2018). Breast density: Why all the fuss?. Clin. Radiol..

[67] Boyd N.F., Rommens J.M., Vogt K., Lee V., Hopper J.L., Yaffe M.J., Paterson A.D. (2005). Mammographic breast density as an intermediate phenotype for breast cancer. Lancet Oncol..

[68] Zdanowski A., Sartor H., Feldt M., Skarping I. (2023). Mammographic density in relation to breast cancer recurrence and survival in women receiving neoadjuvant chemotherapy. Front. Oncol..

[69] Nazari S.S., Mukherjee P. (2018). An overview of mammographic density and its association with breast cancer. Breast Cancer.

[70] Fletcher S.W., Elmore J.G. (2003). Mammographic Screening for Breast Cancer. N. Engl. J. Med..

[71] Woolcott C.G., Koga K., Conroy S.M., Byrne C., Nagata C., Ursin G., Vachon C.M., Yaffe M.J., Pagano I., Maskarinec G. (2012). Mammographic density, parity and age at first birth, and risk of breast cancer: An analysis of four case–control studies. Breast Cancer Res. Treat..

[72] Kerlikowske K., Cook A.J., Buist D.S., Cummings S.R., Vachon C., Vacek P., Miglioretti D.L. (2010). Breast Cancer Risk by Breast Density, Menopause, and Postmenopausal Hormone Therapy Use. J. Clin. Oncol..

[73] Faguy K. (2022). Fibrocystic Breast Changes. Radiol Technol..

[74] Collaborative Group on Hormonal Factors in Breast Cancer (2012). Menarche, menopause, and breast cancer risk: Individual participant meta-analysis, including 118 964 women with breast cancer from 117 epidemiological studies. Lancet Oncol..

[75] Shamliyan T., Wang S.-Y., Virnig B.A., Tuttle T.M., Kane R.L. (2010). Association Between Patient and Tumor Characteristics with Clinical Outcomes in Women with Ductal Carcinoma In Situ. JNCI Monogr..

[76] Miller K. (2003). Estrogen and DNA Damage: The Silent Source of Breast Cancer?. JNCI J. Natl. Cancer Inst..

[77] Barclay J., Ernster V., Kerlikowske K., Grady D., Sickles E.A. (1997). Comparison of Risk Factors for Ductal Carcinoma In Situ and Invasive Breast Cancer. JNCI J. Natl. Cancer Inst..

[78] Ma H., Henderson K.D., Sullivan-Halley J., Duan L., Marshall S.F., Ursin G., Horn-Ross P.L., Largent J., Deapen D.M., Lacey J.V. (2010). Pregnancy-related factors and the risk of breast carcinoma in situand invasive breast cancer among postmenopausal women in the California Teachers Study cohort. Breast Cancer Res..

[79] Merrill R.M., A Folsom J. (2005). Female breast cancer incidence and survival in Utah according to religious preference, 1985–1999. BMC Cancer.

[80] McDaniel S.M., Rumer K.K., Biroc S.L., Metz R.P., Singh M., Porter W., Schedin P. (2006). Remodeling of the Mammary Microenvironment after Lactation Promotes Breast Tumor Cell Metastasis. Am. J. Pathol..

[81] Reunanen N., Kähäri V.M. (2000). Matrix Metalloproteinases in Cancer Cell Invasion. Madame Curie Bioscience Database [Internet].

[82] Gwak H., Woo S.S., Lee E.-S., Park M.H., Lee S., Youn H.J., Park S., Suh I.S., Kim S.H. (2022). Survival of women with pregnancy-associated breast cancer according to clinical characteristics: A propensity score matching study. Medicine.

[83] Chia S.B., Johnson B.J., Hu J., Valença-Pereira F., Chadeau-Hyam M., Guntoro F., Montgomery H., Boorgula M.P., Sreekanth V., Goodspeed A. (2025). Respiratory viral infections awaken metastatic breast cancer cells in lungs. Nature.

[84] Yang S., Zhao M., Jia S. (2023). Macrophage: Key player in the pathogenesis of autoimmune diseases. Front. Immunol..

[85] McLaughlin-Drubin M.E., Munger K. (2008). Viruses associated with human cancer. Biochim. Biophys. Acta (BBA)—Mol. Basis Dis..

[86] Jiang Y., Li Y., Zhu B. (2015). T-cell exhaustion in the tumor microenvironment. Cell Death Dis..

[87] Quinn L.L., Williams L.R., White C., Forrest C., Zuo J., Rowe M. (2016). The Missing Link in Epstein-Barr Virus Immune Evasion: The BDLF3 Gene Induces Ubiquitination and Downregulation of Major Histocompatibility Complex Class I (MHC-I) and MHC-II. J. Virol..

[88] Gabor F., Jahn G., Sedmak D.D., Sinzger C. (2020). In vivo Downregulation of MHC Class I Molecules by HCMV Occurs During All Phases of Viral Replication but Is Not Always Complete. Front. Cell. Infect. Microbiol..

[89] Cornel A.M., Mimpen I.L., Nierkens S. (2020). MHC Class I Downregulation in Cancer: Underlying Mechanisms and Potential Targets for Cancer Immunotherapy. Cancers.

[90] Aloni-Grinstein R., Charni-Natan M., Solomon H., Rotter V. (2018). p53 and the Viral Connection: Back into the Future ^‡^. Cancers.

[91] Keynan Y., Card C.M., McLaren P.J., Dawood M.R., Kasper K., Fowke K.R. (2008). The Role of Regulatory T Cells in Chronic and Acute Viral Infections. Clin. Infect. Dis..

[92] Buchbinder E.I., Desai A. (2016). CTLA-4 and PD-1 Pathways: Similarities, Differences, and Implications of Their Inhibition. Am. J. Clin. Oncol..

[93] Deng Z., Fan T., Xiao C., Tian H., Zheng Y., Li C., He J. (2024). TGF-β signaling in health, disease and therapeutics. Signal Transduct. Target. Ther..

[94] Lebrun J.-J. (2012). The Dual Role of TGFβ in Human Cancer: From Tumor Suppression to Cancer Metastasis. ISRN Mol. Biol..

[95] Yeung K.T., Yang J. (2016). Epithelial–mesenchymal transition in tumor metastasis. Mol. Oncol..

[96] Javelaud D., Alexaki V.I., Dennler S., Mohammad K.S., Guise T.A., Mauviel A. (2011). TGF-β/SMAD/GLI2 Signaling Axis in Cancer Progression and Metastasis. Cancer Res..

[97] Tzavlaki K., Moustakas A. (2020). TGF-β Signaling. Biomolecules.

[98] Shi Y., Massagué J. (2003). Mechanisms of TGF-β Signaling from Cell Membrane to the Nucleus. Cell.

[99] Hill C.S. (2016). Transcriptional Control by the SMADs. Cold Spring Harb. Perspect. Biol..

[100] Shi X., Yang J., Deng S., Xu H., Wu D., Zeng Q., Wang S., Hu T., Wu F., Zhou H. (2022). TGF-β signaling in the tumor metabolic microenvironment and targeted therapies. J. Hematol. Oncol..

[101] Pham A.H., Mitchell J., Botto S., Pryke K.M., DeFilippis V.R., Hancock M.H. (2021). Human cytomegalovirus blocks canonical TGFβ signaling during lytic infection to limit induction of type I interferons. PLoS Pathog..

[102] Silva J.d.M., Alves C.E.d.C., Pontes G.S. (2024). Epstein-Barr virus: The mastermind of immune chaos. Front. Immunol..

[103] Mahmud J., Geiler B.W., Biswas J., Miller M.J., Myers J.E., Matthews S.M., Wass A.B., O’Connor C.M., Chan G.C. (2023). Virion-associated US28 rapidly modulates Akt activity to suppress HCMV lytic replication in monocytes. bioRxiv.

[104] Jasek-Gajda E., Jurkowska H., Jasińska M., Lis G.J. (2020). Targeting the MAPK/ERK and PI3K/AKT Signaling Pathways Affects NRF2, Trx and GSH Antioxidant Systems in Leukemia Cells. Antioxidants.

[105] Paulus C., Nevels M. (2009). The Human Cytomegalovirus Major Immediate-Early Proteins as Antagonists of Intrinsic and Innate Antiviral Host Responses. Viruses.

[106] Dunn E.F., Connor J.H. (2012). HijAkt: The PI3K/Akt pathway in virus replication and pathogenesis. Prog. Mol. Biol. Transl. Sci..

[107] Roy S., Arav-Boger R. (2014). New Cell-Signaling Pathways for Controlling Cytomegalovirus Replication. Am. J. Transplant..

[108] Tang Y.-L., Wang S.-S., Jiang J., Liang X.-H. (2015). Links between cancer stem cells and epithelial– mesenchymal transition. OncoTargets Ther..

[109] Furler R.L., Nixon D.F., Brantner C.A., Popratiloff A., Uittenbogaart C.H. (2018). TGF-β Sustains Tumor Progression through Biochemical and Mechanical Signal Transduction. Cancers.

[110] Krstic J., Santibanez J.F. (2014). Transforming Growth Factor-Beta and Matrix Metalloproteinases: Functional Interactions in Tumor Stroma-Infiltrating Myeloid Cells. Sci. World J..

[111] Biernacka A., Dobaczewski M., Frangogiannis N.G. (2011). TGF-β signaling in fibrosis. Growth Factors.

[112] Yuan S., Zhu W., Wang Y., Yu L. (2025). The Function of the TGFβ Signaling Pathway in Connective Tissue Diseases: From Biology to Clinical Application. J. Inflamm. Res..

[113] Kim Y., Stolarska M.A., Othmer H.G. (2011). The role of the microenvironment in tumor growth and invasion. Prog. Biophys. Mol. Biol..

[114] Lieberman P.M. (2016). Epigenetics and Genetics of Viral Latency. Cell Host Microbe.

[115] Murata T., Sugimoto A., Inagaki T., Yanagi Y., Watanabe T., Sato Y., Kimura H. (2021). Molecular Basis of Epstein–Barr Virus Latency Establishment and Lytic Reactivation. Viruses.

[116] Baasch S., Ruzsics Z., Henneke P. (2020). Cytomegaloviruses and Macrophages—Friends and Foes From Early on?. Front. Immunol..

[117] Chen L., Deng H., Cui H., Fang J., Zuo Z., Deng J., Li Y., Wang X., Zhao L. (2017). Inflammatory responses and inflammation-associated diseases in organs. Oncotarget.

[118] Lindau D., Gielen P., Kroesen M., Wesseling P., Adema G.J. (2012). The immunosuppressive tumour network: Myeloid-derived suppressor cells, regulatory T cells and natural killer T cells. Immunology.

[119] Varani S., Landini M.P. (2011). Cytomegalovirus-induced immunopathology and its clinical consequences. Herpesviridae.

[120] Alsaadawe M., Radman B.A., Hu L., Long J., Luo Q., Tan C., Amirat H.S., Alsaadawi M., Lyu X. (2025). From Viral Infection to Malignancy: The Dual Threat of EBV and COVID-19 in Cancer Development. Viruses.

[121] Ortmann B.M. (2024). Hypoxia-inducible factor in cancer: From pathway regulation to therapeutic opportunity. BMJ Oncol..

[122] Scott R.S. (2017). Epstein–Barr virus: A master epigenetic manipulator. Curr. Opin. Virol..

[123] Wei H., Zhou M.-M. (2009). Viral-encoded enzymes that target host chromatin functions. Biochim. Biophys. Acta (BBA)—Gene Regul. Mech..

[124] Sadanandam A., Lal A., Benz S.C., Eppenberger-Castori S., Scott G., Gray J.W., Spellman P., Waldman F., Benz C.C. (2012). Genomic aberrations in normal tissue adjacent to HER2-amplified breast cancers: Field cancerization or contaminating tumor cells?. Breast Cancer Res. Treat..

[125] Heaphy C.M., Bisoffi M., Fordyce C.A., Haaland C.M., Hines W.C., Joste N.E., Griffith J.K. (2006). Telomere DNA content and allelic imbalance demonstrate field cancerization in histologically normal tissue adjacent to breast tumors. Int. J. Cancer.

[126] Aran D., Camarda R., Odegaard J., Paik H., Oskotsky B., Krings G., Goga A., Sirota M., Butte A.J. (2017). Comprehensive analysis of normal adjacent to tumor transcriptomes. Nat. Commun..

[127] Huang X., Stern D.F., Zhao H. (2016). Transcriptional Profiles from Paired Normal Samples Offer Complementary Information on Cancer Patient Survival—Evidence from TCGA Pan-Cancer Data. Sci. Rep..

[128] Trujillo K.A., Hines W.C., Vargas K.M., Jones A.C., Joste N.E., Bisoffi M., Griffith J.K. (2011). Breast Field Cancerization: Isolation and Comparison of Telomerase-Expressing Cells in Tumor and Tumor Adjacent, Histologically Normal Breast Tissue. Mol. Cancer Res..

[129] Lewis C.M., Cler L.R., Bu D.-W., ZöcHbauer-MülLer S., Milchgrub S., Naftalis E.Z., Leitch A.M., Minna J.D., Euhus D.M. (2005). Promoter Hypermethylation in Benign Breast Epithelium in Relation to Predicted Breast Cancer Risk. Clin. Cancer Res..

[130] Lehmann U., Länger F., Feist H., Glöckner S., Hasemeier B., Kreipe H. (2002). Quantitative Assessment of Promoter Hypermethylation during Breast Cancer Development. Am. J. Pathol..

[131] Forsberg L.A., Rasi C., Pekar G., Davies H., Piotrowski A., Absher D., Razzaghian H.R., Ambicka A., Halaszka K., Przewoźnik M. (2015). Signatures of post-zygotic structural genetic aberrations in the cells of histologically normal breast tissue that can predispose to sporadic breast cancer. Genome Res..

[132] Försti A., Louhelainen J., Söderberg M., Wijkström H., Hemminki K. (2001). Loss of heterozygosity in tumour-adjacent normal tissue of breast and bladder cancer. Eur. J. Cancer.

[133] Gadaleta E., Fourgoux P., Pirró S., Thorn G.J., Nelan R., Ironside A., Rajeeve V., Cutillas P.R., Lobley A.E., Wang J. (2020). Characterization of four subtypes in morphologically normal tissue excised proximal and distal to breast cancer. npj Breast Cancer.

[134] Graham K., Morenas A.d.L., Tripathi A., King C., Kavanah M., Mendez J., Stone M., Slama J., Miller M., Antoine G. (2010). Gene expression in histologically normal epithelium from breast cancer patients and from cancer-free prophylactic mastectomy patients shares a similar profile. Br. J. Cancer.

[135] Larson P., Morenas A.d.L., Cerda S., Bennett S., Cupples L., Rosenberg C. (2006). Quantitative analysis of allele imbalance supports atypical ductal hyperplasia lesions as direct breast cancer precursors. J. Pathol..

[136] Román-Pérez E., Casbas-Hernández P., Pirone J.R., Rein J., Carey L.A., Lubet R.A., Mani S.A., Amos K.D., Troester M.A. (2012). Gene expression in extratumoral microenvironment predicts clinical outcome in breast cancer patients. Breast Cancer Res..

[137] A Troester M., A Hoadley K., D’aRcy M., Cherniack A.D., Stewart C., Koboldt D.C., Robertson A.G., Mahurkar S., Shen H., Wilkerson M.D. (2016). DNA defects, epigenetics, and gene expression in cancer-adjacent breast: A study from The Cancer Genome Atlas. npj Breast Cancer.

[138] Trujillo K.A., Heaphy C.M., Mai M., Vargas K.M., Jones A.C., Vo P., Butler K.S., Joste N.E., Bisoffi M., Griffith J.K. (2010). Markers of fibrosis and epithelial to mesenchymal transition demonstrate field cancerization in histologically normal tissue adjacent to breast tumors. Int. J. Cancer.

[139] Klimov S., Miligy I.M., Gertych A., Jiang Y., Toss M.S., Rida P., Ellis I.O., Green A., Krishnamurti U., Rakha E.A. (2019). A whole slide image-based machine learning approach to predict ductal carcinoma in situ (DCIS) recurrence risk. Breast Cancer Res..

[140] Lengauer C., Kinzler K.W., Vogelstein B. (1998). Genetic instabilities in human cancers. Nature.

[141] Ellsworth D.L., Ellsworth R.E., Love B., Deyarmin B., Lubert S.M., Mittal V., Shriver C.D. (2004). Genomic patterns of allelic imbalance in disease free tissue adjacent to primary breast carcinomas. Breast Cancer Res. Treat..

[142] Larson P.S., Morenas A.D.L., Cupples L.A., Huang K., Rosenberg C.L. (1998). Genetically abnormal clones in histologically normal breast tissue. Am. J. Pathol..

[143] Larson P.S., Morenas A.d.L., Bennett S.R., Cupples L.A., Rosenberg C.L. (2002). Loss of Heterozygosity or Allele Imbalance in Histologically Normal Breast Epithelium Is Distinct from Loss of Heterozygosity or Allele Imbalance in Co-Existing Carcinomas. Am. J. Pathol..

[144] Li Z., Moore D.H., Meng Z.H., Ljung B.-M., Gray J.W., Dairkee S.H. (2002). Increased risk of local recurrence is associated with allelic loss in normal lobules of breast cancer patients. Cancer Res..

[145] Kreiger N., Sloan M., Cotterchio M., Kirsh V. (1999). The risk of breast cancer following reproductive surgery. Eur. J. Cancer.

[146] Askenase P.W. (2021). Exosomes provide unappreciated carrier effects that assist transfers of their miRNAs to targeted cells; I. They are ‘The Elephant in the Room’. RNA Biol..

[147] Yue W., Santen R., Wang J.-P., Li Y., Verderame M., Bocchinfuso W., Korach K., Devanesan P., Todorovic R., Rogan E. (2003). Genotoxic metabolites of estradiol in breast: Potential mechanism of estradiol induced carcinogenesis. J. Steroid Biochem. Mol. Biol..

[148] Ellsworth D.L., Ellsworth R.E., Liebman M.N., Hooke J.A., Shriver C.D. (2004). Genomic instability in histologically normal breast tissues: Implications for carcinogenesis. Lancet Oncol..

[149] Teo N.B., Shoker B., Jarvis C., Martin L., Sloane J., Holcombe C. (2003). Angiogenesis and invasive recurrence in ductal carcinoma in situ of the breast. Eur. J. Cancer.

[150] Mittal K., Toss M.S., Wei G., Kaur J., Choi D.H., Melton B.D., Osan R.M., Miligy I.M., Green A.R., Janssen E.A. (2020). A Quantitative Centrosomal Amplification Score Predicts Local Recurrence of Ductal Carcinoma In Situ. Clin. Cancer Res..

[151] Denu R.A., Zasadil L.M., Kanugh C., Laffin J., Weaver B.A., Burkard M.E. (2016). Centrosome amplification induces high grade features and is prognostic of worse outcomes in breast cancer. BMC Cancer.

[152] Nojima H. (2004). G1 and S-phase checkpoints, chromosome instability, and cancer. Methods Mol. Biol..

[153] Sun X., Ma X., Wang J., Zhao Y., Wang Y., Bihl J.C., Chen Y., Jiang C. (2017). Glioma stem cells-derived exosomes promote the angiogenic ability of endothelial cells through miR-21/VEGF signal. Oncotarget.

[154] King H.W., Michael M.Z., Gleadle J.M. (2012). Hypoxic enhancement of exosome release by breast cancer cells. BMC Cancer.

[155] Lucero R., Zappulli V., Sammarco A., Murillo O.D., Cheah P.S., Srinivasan S., Tai E., Ting D.T., Wei Z., Roth M.E. (2020). Glioma-Derived miRNA-Containing Extracellular Vesicles Induce Angiogenesis by Reprogramming Brain Endothelial Cells. Cell Rep..

[156] Yang S., Kuo C., Bisi J.E., Kim M.K. (2002). PML-dependent apoptosis after DNA damage is regulated by the checkpoint kinase hCds1/Chk2. Nat. Cell Biol..

[157] Muse M.E., Titus A.J., Salas L.A., Wilkins O.M., Mullen C., Gregory K.J., Schneider S.S., Crisi G.M., Jawale R.M., Otis C.N. (2020). Enrichment of CpG island shore region hypermethylation in epigenetic breast field cancerization. Epigenetics.

[158] Yan P.S., Venkataramu C., Ibrahim A., Liu J.C., Shen R.Z., Diaz N.M., Centeno B., Weber F., Leu Y.-W., Shapiro C.L. (2006). Mapping Geographic Zones of Cancer Risk with Epigenetic Biomarkers in Normal Breast Tissue. Clin. Cancer Res..

[159] Gao Y., Widschwendter M., Teschendorff A.E. (2018). DNA Methylation Patterns in Normal Tissue Correlate more Strongly with Breast Cancer Status than Copy-Number Variants. eBioMedicine.

[160] Mani S.A., Guo W., Liao M.-J., Eaton E.N., Ayyanan A., Zhou A.Y., Brooks M., Reinhard F., Zhang C.C., Shipitsin M. (2008). The Epithelial-Mesenchymal Transition Generates Cells with Properties of Stem Cells. Cell.

[161] Lebya K., Garcia-Smith R., Swaminathan R., Jones A., Russell J., Joste N., Bisoffi M., Trujillo K. (2017). Towards a personalized surgical margin for breast conserving surgery—Implications of field cancerization in local recurrence. J. Surg. Oncol..

[162] Saliminejad K., Khorram Khorshid H.R., Soleymani Fard S., Ghaffari S.H. (2019). An overview of microRNAs: Biology, functions, therapeutics, and analysis methods. J. Cell. Physiol..

[163] Shen S., Sun Q., Liang Z., Cui X., Ren X., Chen H., Zhang X., Zhou Y. (2014). A Prognostic Model of Triple-Negative Breast Cancer Based on miR-27b-3p and Node Status. PLoS ONE.

[164] Mathe A., Scott R.J., Avery-Kiejda K.A. (2015). miRNAs and Other Epigenetic Changes as Biomarkers in Triple Negative Breast Cancer. Int. J. Mol. Sci..

[165] McKee A.M., Kirkup B.M., Madgwick M., Fowler W.J., Price C.A., Dreger S.A., Ansorge R., Makin K.A., Caim S., Le Gall G. (2021). Antibiotic-induced disturbances of the gut microbiota result in accelerated breast tumor growth. iScience.

[166] Ingman W.V. (2019). The Gut Microbiome: A New Player in Breast Cancer Metastasis. Cancer Res..

[167] Urbaniak C., Gloor G.B., Brackstone M., Scott L., Tangney M., Reid G. (2016). The Microbiota of Breast Tissue and Its Association with Breast Cancer. Appl. Environ. Microbiol..

[168] Xuan C., Shamonki J.M., Chung A., DiNome M.L., Chung M., Sieling P.A., Lee D.J. (2014). Microbial Dysbiosis Is Associated with Human Breast Cancer. PLoS ONE.

[169] Banerjee S., Tian T., Wei Z., Shih N., Feldman M.D., Peck K.N., DeMichele A.M., Alwine J.C., Robertson E.S. (2018). Distinct Microbial Signatures Associated with Different Breast Cancer Types. Front. Microbiol..

[170] Becker A., Thakur B.K., Weiss J.M., Kim H.S., Peinado H., Lyden D. (2016). Extracellular Vesicles in Cancer: Cell-to-Cell Mediators of Metastasis. Cancer Cell.

[171] Li C., Zhou T., Chen J., Li R., Chen H., Luo S., Chen D., Cai C., Li W. (2022). The role of Exosomal miRNAs in cancer. J. Transl. Med..

[172] Guay C., Regazzi R. (2017). Exosomes as new players in metabolic organ cross-talk. Diabetes, Obes. Metab..

[173] Whiteside T.L. (2018). Exosome and mesenchymal stem cell cross-talk in the tumor microenvironment. Semin. Immunol..

[174] Fang T., Lv H., Lv G., Li T., Wang C., Han Q., Yu L., Su B., Guo L., Huang S. (2018). Tumor-derived exosomal miR-1247-3p induces cancer-associated fibroblast activation to foster lung metastasis of liver cancer. Nat. Commun..

[175] Tang X., Hou Y., Yang G., Wang X., Tang S., Du Y.-E., Yang L., Yu T., Zhang H., Zhou M. (2015). Stromal miR-200s contribute to breast cancer cell invasion through CAF activation and ECM remodeling. Cell Death Differ..

[176] Liu Y., Li X., Zhang Y., Wang H., Rong X., Peng J., He L., Peng Y. (2019). An miR-340-5p-macrophage feedback loop modulates the progression and tumor microenvironment of glioblastoma multiforme. Oncogene.

[177] De Silva N., Samblas M., Martínez J.A., I Milagro F. (2018). Effects of exosomes from LPS-activated macrophages on adipocyte gene expression, differentiation, and insulin-dependent glucose uptake. J. Physiol. Biochem..

[178] Rodríguez-Martínez A., de Miguel-Pérez D., Ortega F.G., García-Puche J.L., Robles-Fernández I., Exposito J., Martorell-Marugan J., Carmona-Sáez P., Garrido-Navas M.d.C., Rolfo C. (2019). Exosomal miRNA profile as complementary tool in the diagnostic and prediction of treatment response in localized breast cancer under neoadjuvant chemotherapy. Breast Cancer Res..

[179] Tai Y.-L., Chen K.-C., Hsieh J.-T., Shen T.-L. (2018). Exosomes in cancer development and clinical applications. Cancer Sci..

[180] Zhao Z., Yang S., Zhou A., Li X., Fang R., Zhang S., Zhao G., Li P. (2021). Small Extracellular Vesicles in the Development, Diagnosis, and Possible Therapeutic Application of Esophageal Squamous Cell Carcinoma. Front. Oncol..

[181] Yan W., Wu X., Zhou W., Fong M.Y., Cao M., Liu J., Liu X., Chen C.-H., Fadare O., Pizzo D.P. (2018). Cancer-cell-secreted exosomal miR-105 promotes tumour growth through the MYC-dependent metabolic reprogramming of stromal cells. Nat. Cell Biol..

[182] Petrova V., Annicchiarico-Petruzzelli M., Melino G., Amelio I. (2018). The hypoxic tumour microenvironment. Oncogenesis.

[183] Pontecorvi G., Bellenghi M., Puglisi R., Carè A., Mattia G. (2020). Tumor-derived extracellular vesicles and microRNAs: Functional roles, diagnostic, prognostic and therapeutic options. Cytokine Growth Factor Rev..

[184] Zhou Y., Ren H., Dai B., Li J., Shang L., Huang J., Shi X. (2018). Hepatocellular carcinoma-derived exosomal miRNA-21 contributes to tumor progression by converting hepatocyte stellate cells to cancer-associated fibroblasts. J. Exp. Clin. Cancer Res..

[185] Tan S., Xia L., Yi P., Han Y., Tang L., Pan Q., Tian Y., Rao S., Oyang L., Liang J. (2020). Exosomal miRNAs in tumor microenvironment. J. Exp. Clin. Cancer Res..

[186] Mei W., Tabrizi S.F., Godina C., Lovisa A.F., Isaksson K., Jernström H., Tavazoie S.F. (2024). A commonly inherited human PCSK9 germline variant drives breast cancer metastasis via LRP1 receptor. Cell.

[187] Webber J., Steadman R., Mason M.D., Tabi Z., Clayton A. (2010). Cancer Exosomes Trigger Fibroblast to Myofibroblast Differentiation. Cancer Res..

[188] Fattore L., Ruggiero C.F., Pisanu M.E., Liguoro D., Cerri A., Costantini S., Capone F., Acunzo M., Romano G., Nigita G. (2019). Reprogramming miRNAs global expression orchestrates development of drug resistance in BRAF mutated melanoma. Cell Death Differ..

[189] Fernando M.R., Jiang C., Krzyzanowski G.D., Ryan W.L. (2017). New evidence that a large proportion of human blood plasma cell-free DNA is localized in exosomes. PLoS ONE.

[190] Ruksha T., Palkina N. (2025). Role of exosomes in transforming growth factor-β-mediated cancer cell plasticity and drug resistance. Explor. Target. Antitumor Ther..

[191] Khan S., Jutzy J.M.S., Aspe J.R., McGregor D.W., Neidigh J.W., Wall N.R. (2010). Survivin is released from cancer cells via exosomes. Apoptosis.

[192] Khan S., Bennit H.F., Wall N.R. (2014). The emerging role of exosomes in survivin secretion. Histol. Histopathol..

[193] Wu S., Turner K.M., Nguyen N., Raviram R., Erb M., Santini J., Luebeck J., Rajkumar U., Diao Y., Li B. (2019). Circular ecDNA promotes accessible chromatin and high oncogene expression. Nature.

[194] Turner K.M., Deshpande V., Beyter D., Koga T., Rusert J., Lee C., Li B., Arden K., Ren B., Nathanson D.A. (2017). Extrachromosomal oncogene amplification drives tumour evolution and genetic heterogeneity. Nature.

[195] Pecorino L.T., Verhaak R.G., Henssen A., Mischel P.S. (2022). Extrachromosomal DNA (ecDNA): An origin of tumor heterogeneity, genomic remodeling, and drug resistance. Biochem. Soc. Trans..

[196] Elzanowska J., Semira C., Costa-Silva B. (2020). DNA in extracellular vesicles: Biological and clinical aspects. Mol. Oncol..

[197] Moroishi T., Hayashi T., Pan W.-W., Fujita Y., Holt M.V., Qin J., Carson D.A., Guan K.-L. (2016). The Hippo Pathway Kinases LATS1/2 Suppress Cancer Immunity. Cell.

[198] Muhsin-Sharafaldine M.-R., Saunderson S.C., Dunn A.C., Faed J.M., Kleffmann T., McLellan A.D. (2016). Procoagulant and immunogenic properties of melanoma exosomes, microvesicles and apoptotic vesicles. Oncotarget.

[199] Yang Y., Li C.-W., Chan L.-C., Wei Y., Hsu J.-M., Xia W., Cha J.-H., Hou J., Hsu J.L., Sun L. (2018). Exosomal PD-L1 harbors active defense function to suppress T cell killing of breast cancer cells and promote tumor growth. Cell Res..

[200] Zhou M., Chen J., Zhou L., Chen W., Ding G., Cao L. (2014). Pancreatic cancer derived exosomes regulate the expression of TLR4 in dendritic cells via miR-203. Cell. Immunol..

